# Absorption characteristics and *in vivo* behavior of astaxanthin isomers: insights from the administration of highly purified (all-*E*)-, (9*Z*)-, and (13*Z*)-astaxanthin in male mice

**DOI:** 10.1039/d5ra06060e

**Published:** 2025-11-10

**Authors:** Antara Ghosh, Yoshiharu Sawada, Kentaro Takahama, Yasuhiro Nishida, Masaki Honda

**Affiliations:** a Department of Chemistry, Faculty of Science & Technology, Meijo University 1-501 Shiogamaguchi, Tempaku-ku Nagoya Aichi 468-8502 Japan honda@meijo-u.ac.jp; b Technical Center, Nagoya University Furo-cho Nagoya Aichi 464-8601 Japan; c Fuji Chemical Industries, Co., Ltd 55 Yokohoonji, Kamiich-machi Nakaniikawa-gun Toyama 930-0405 Japan octopacy1978@gmail.com; d Graduate School of Environmental and Human Sciences, Meijo University 1-501 Shiogamaguchi, Tempaku-ku Nagoya Aichi 468-8502 Japan

## Abstract

Astaxanthin, a xanthophyll carotenoid with potent antioxidant properties, exists in multiple geometrical isomers whose differences in bioavailability, *in vivo* stability, and isomerization behavior remain insufficiently characterized. Here, we conducted a comprehensive comparative analysis of the pharmacokinetics of purified (all-*E*)-, (9*Z*)-, and (13*Z*)-astaxanthin isomers following oral and intravenous administration in ICR male mice. Using both non-compartmental and population pharmacokinetic (PopPK) modeling, we quantified isomer-specific parameters including clearance (CL), bioavailability (*F*), and absorption lag time (*T*_lag_). Among the isomers, (13*Z*)-astaxanthin exhibited superior systemic exposure and hepatic accumulation, with markedly lower clearance and higher oral bioavailability than the 9*Z*- and all-*E*-isomers. In contrast, (9*Z*)-astaxanthin demonstrated rapid elimination and extensive *in vivo* isomerization, possibly associated with enzymatic metabolism during intestinal absorption. Molecular docking simulations revealed stronger binding affinities of (13*Z*)-astaxanthin to key transport proteins, such as apolipoprotein AI (ApoA-I) and serum albumin (SA), supporting its prolonged systemic retention. These findings not only confirm that geometric isomerism significantly influences the pharmacokinetic behavior of astaxanthin but also reconcile conflicting literature regarding the bioavailability of *Z*-isomer-rich carotenoids. Importantly, our data underscore the need for isomer-specific formulation strategies and suggest that (13*Z*)-astaxanthin is a promising candidate for enhanced delivery in nutraceutical and therapeutic applications.

## Abstract 2

(13*Z*)-Astaxanthin exhibits significantly higher bioavailability, systemic retention, and hepatic accumulation than the (all-*E*)- and (9*Z*)-isomers, highlighting its potential for nutraceutical applications.

## Graphical abstract

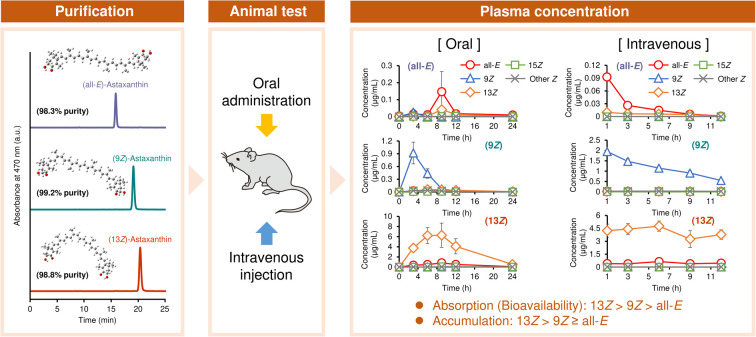

## 1. Introduction

Astaxanthin is a natural xanthophyll carotenoid found in various microorganisms and marine animals.^1,2^ Traditionally, this carotenoid has been utilized in aquaculture to impart pigmentation to salmonids and shrimps.^3,4^ More recently, its potent antioxidant properties and diverse biological activities have attracted attention, expanding its use in the food industry.^5,6^ However, the high crystallinity and hydrophobicity of astaxanthin limit its bioavailability, which remains a major challenge for practical applications.^7,8^ To overcome this issue, several water-soluble formulation strategies, such as emulsification and nanoparticle techniques, have been examined to enhance astaxanthin bioavailability; however, their impact has remained limited.^9–11^

In nature, astaxanthin is predominantly found in the all-*E*-configuration; however, isomerization of certain double bonds to the *Z*-configuration has been shown to markedly enhance its bioavailability.^12–16^ Furthermore, several recent studies have shown that astaxanthin *Z*-isomers exhibit higher biological activities, such as anti-elastase, anti-inflammatory, and anti-obesity activities, than the all-*E*-isomer.^17–19^ Therefore, the isomerization of astaxanthin to its *Z*-isomer has recently garnered considerable attention as a novel strategy to improve its bioavailability as well as its health promotion effects.

Astaxanthin possesses multiple geometric isomers, with at least five *Z*-isomers identified in processed foods and animal bodies.^14–16,20^ In most cases, the main *Z*-isomers contained in them are the 9*Z*- and 13*Z*-isomers. However, the bioavailability and pharmacokinetics of each isomer remain unclear. Although the superior bioavailability of astaxanthin *Z*-isomers has been demonstrated in various animal studies, these evaluations relied on mixtures predominantly containing the 9*Z*- and 13*Z*-isomers, making it difficult to determine which specific isomer is responsible for the enhanced absorption.^14–16,21,22^ Moreover, even when diets enriched in (9*Z*)-astaxanthin is administered, its concentration remains considerably low in the bodies of many animals.^14,21,22^ To accurately assess the bioavailability and pharmacokinetics of astaxanthin *Z*-isomers, conducting oral administration experiments using high-purity isomer preparations is essential. Additionally, to precisely determine the pharmacokinetics, data must be obtained from intravenous administration experiments to exclude the confounding effects of isomerization and degradation of astaxanthin during the digestive process following oral administration.^23,24^

This study aimed to distinguish the absorption characteristics and pharmacokinetics of astaxanthin isomers by administering high-purity astaxanthin isomers orally and intravenously to mice. The evaluation focused on the all-*E*-, 9*Z*- and 13*Z*-isomers, the predominant isomers present in foodstuffs and within the animal bodies.^14–16,20^ Obtaining high-purity (9*Z*)- and (13*Z*)-astaxanthin has been challenging so far because they are rare in nature and difficult to isolate by crystallization or chromatography.^12,25^ We recently established a method to efficiently obtain the 9*Z*- and 13*Z*-isomers from the readily available all-*E*-isomer through a heat-induced process,^14,17,26^ as well as a technique to clearly separate these isomers using reverse-phase chromatography.^20^ By utilizing these techniques, we successfully prepared large quantities of high-purity (9*Z*)- and (13*Z*)-astaxanthin (>98% purity). This study offers a detailed assessment of the bioavailability, *in vivo* stability, and isomerization behavior of astaxanthin isomers with the aim of improving their application in food and health products, and ultimately enhancing their therapeutic effects.

## 2. Materials and methods

### 2.1. Reagents

High-purity (all-*E*)-astaxanthin crystals (>98% purity) was provided from Fuji Chemical Co., Ltd (Toyama, Japan). High-performance liquid chromatography (HPLC)-grade organic solvents (acetone, ethyl acetate, and hexane), *N*,*N*-dimethylacetamide, polyethylene glycol 400, and soybean oil were purchased from FUJIFILM Wako Pure Chemical Corporation (Osaka, Japan). Analytical-grade organic solvents [methanol, acetone, and dichloromethane (CH_2_Cl_2_)] and butylated hydroxytoluene (BHT) were obtained from Kanto Chemical Co., Inc. (Tokyo, Japan), and chloroform-d_1_ (CDCl_3_) for NMR analysis was purchased from Isotec Inc. (Miamisburg, OH, USA). Xylazine hydrochloride (Seractal) and ketamine hydrochloride (Ketalar) were purchased from Bayer AG (Leverkusen, Germany) and Daiichi Sankyo Propharma Co. Ltd (Tokyo, Japan), respectively.

### 2.2. Thermal isomerization and purification of astaxanthin isomers

A *Z*-isomer-rich astaxanthin mixture was prepared from the all-*E*-isomer by thermal treatment following previously reported procedures.^14,17^ In brief, the (all-*E*)-astaxanthin crystals were dissolved in CH_2_Cl_2_ at a concentration of 10 mg mL^−1^. The solution was transferred to a stainless steel vessel (TPR3-VS2-120; SUS 316, Taiatsu Garasu Kogyo Co., Ltd, Tokyo, Japan), pressurized to 5 MPa with nitrogen, and then heated at 180 °C for 15 min in an oil bath (SOS-183D, Sansho Co. Ltd, Tokyo). After heating, the vessel was immediately cooled in ice water, and CH_2_Cl_2_ was removed using an evaporator (EYELA N-1300 system; Tokyo Rikakikai Co., Ltd, Tokyo, Japan). The obtained solid was suspended in a small amount of methanol, followed by filtration through a 0.22 μm polytetrafluoroethylene (PTFE) membrane filter (Osaka Chemical Co., Ltd, Osaka, Japan) to remove insoluble substances (mainly the all-*E*-isomer crystals). The obtained filtrate was rich in astaxanthin *Z*-isomers, particularly the 9*Z*- and 13*Z*-isomers. The (9*Z*)- and (13*Z*)-astaxanthin isomers were separated from the *Z*-isomer-rich astaxanthin mixture using preparative HPLC (FRC-10A; Shimadzu, Kyoto, Japan). The separation was performed using a COSMOSIL 5C18-MS-II column (400 mm × 10 mm i.d., 5 μm particle size; Nacalai Tesque Inc., Kyoto, Japan) as the stationary phase.^20^ The mobile phase consisted of a methanol/water mixture (92.5 : 7.5, v/v) with a flow rate of 3.0 mL min^−1^, and the column temperature was maintained at 30 °C. From approximately 1.5 g of crystalline all-*E*-isomer of astaxanthin, 41.0 mg of the 9*Z*-isomer and 25.0 mg of the 13*Z*-isomer were obtained through this process. The purified 9*Z*- and 13*Z*-isomers were immediately dissolved in soybean oil for the oral administration or a mixture of *N*,*N*-dimethylacetamide and polyethylene glycol 400 (1 : 1, v/v) for the intravenous injection,^14,24^ and stored at −80 °C under dark condition until just before the administration.

### 2.3. Animal treatment

All animal experiments were performed in the I Tech Lab. Co., Ltd (Gifu, Japan). The study protocol was approved by the Institutional Animal Care and Use Committee of the company (approval numbers: ITL-25-MO-385 for the oral administration test and ITL-25-MV-386 for the intravenous injection test). Slc:ICR (ICR) male mice aged 7–8 weeks, supplied by Japan SLC Inc. (Shizuoka, Japan), were used in this study. The body weight of the mice at the beginning of the experiments ranged from 42.8 to 52.3 g. The mice were maintained under stable laboratory conditions (temperature: 18–28 °C; humidity: 30–80%; 12 h light/dark cycle) and provided with drinking water and a standard laboratory diet (MF; Oriental Yeast Co., Ltd, Tokyo, Japan) throughout the acclimation and testing periods. After an 11-day acclimation period, single oral administration and intravenous injection tests were conducted. For the oral administration test, 15 mice were randomly assigned to four groups: control (placebo; soybean oil administered), (all-*E*)-astaxanthin administered (*n* = 4), (9*Z*)-astaxanthin administered (*n* = 4), and (13*Z*)-astaxanthin administered (*n* = 4). Each astaxanthin isomer was administered to mice at a dose of 100 mg per kg body weight. It should be noted that this oral dose exceeds typical dietary exposure levels; however, it was intentionally selected to ensure sufficient systemic concentrations for reliable quantification of each isomer. This dosing strategy is in line with previous pharmacokinetic studies of carotenoids in rodents.^14,15^ Blood samples were collected before and 3, 6, 9, 12, and 24 h after the administration of the test diets. Fecal samples were collected 6–24 h after administering astaxanthin. In the intravenous injection test, 15 mice were randomly assigned to four groups: control (placebo; solvent intravenously injected), (all-*E*)-astaxanthin intravenously injected (*n* = 4), (9*Z*)-astaxanthin intravenously injected (*n* = 4), and (13*Z*)-astaxanthin intravenously injected (*n* = 4). Each astaxanthin isomer was intravenously injected into mice at a dose of 10 mg per kg body weight.^24^ Blood samples were collected before and 1, 3, 6, 9, and 12 h after injecting the test samples. After these tests, the mice were sacrificed by exsanguination under anesthesia (ketamine hydrochloride and xylazine hydrochloride), and the liver was collected. Plasma samples were prepared from heparin-treated blood by centrifugation. The collected plasma, liver, and fecal samples were immediately stored at −80 °C under dark condition until just before astaxanthin extraction.

### 2.4. Extraction of astaxanthin isomers

Astaxanthin isomers in the plasma, liver, and fecal samples were extracted according to previous reports.^14,15^ In brief, astaxanthin isomers in plasma were extracted using hexane containing 0.01% BHT. An equal volume of methanol was added to each plasma sample to precipitate the proteins prior to hexane extraction. The extraction was performed three times using a vortex mixer for 30 s. After separating the hexane and aqueous layers by centrifugation, the hexane layer was carefully collected and removed using an evaporator. The obtained solid was dissolved in a mixture of hexane and ethyl acetate (9 : 1), filtered through a 0.22 μm PTFE membrane filter, and analyzed using HPLC. Astaxanthin isomers in liver and fecal samples were extracted using acetone containing 0.01% BHT. The samples were ultrasonicated at 80 W and 38 kHz for 30 min in ice water (∼5 °C) to facilitate extraction. The extraction residue was separated from the extraction liquid using a 0.22 μm PTFE membrane filter. The filtrate containing astaxanthin isomers was concentrated to dryness using an evaporator. Finally, the residue was dissolved in a mixture of hexane and ethyl acetate (9 : 1), filtered through a 0.22 μm PTFE membrane filter, and analyzed using HPLC. To minimize the isomerization and degradation of astaxanthin during the extraction process, heat and light exposure were carefully controlled. HPLC analysis of samples was performed within 24 h of extraction, and samples were stored at −80 °C until just prior to analysis.

### 2.5. Analysis of astaxanthin isomers

Astaxanthin isomers in the plasma, liver, and fecal samples were analyzed using normal-phase HPLC equipped with an SPD-M20A photodiode array detector (Shimadzu Corp., Kyoto, Japan), following previously reported methods.^14,15,27^ Normal-phase chromatography was selected because it provided superior separation not only among astaxanthin isomers but also between astaxanthin and other carotenoids in plasma samples and *Haematococcus* extracts, compared with conventional reverse-phase analysis. The analysis was performed under the following conditions: a stationary phase comprising two serially connected Phenomenex silica gel columns (Luna 5 μm Silica (2), 150 mm × 4.6 mm, 100 Å; Phenomenex Inc., Torrance, CA, USA); a mobile phase consisting of a mixture of hexane, ethyl acetate, and acetone (75 : 23 : 2, v/v/v); a mobile phase flow rate of 1.2 mL min^−1^; a column temperature of 40 °C; and a detection wavelength of 470 nm. Astaxanthin isomer peaks were identified by comparing HPLC retention times, absorption spectra, mass spectra, and ^1^H-NMR spectra, as previously reported.^26–30^ Mass spectrometry analysis was conducted on a Shimadzu LCMS-2050 mass spectrometer (Shimadzu Corp., Kyoto, Japan), configured with a Dual Ion Source, enabling simultaneous atmospheric pressure chemical ionization and electrospray ionization in positive ion mode.^27^ Nitrogen was employed as the nebulizing and drying gases at flow rates of 2.0 and 5.0 L min^−1^, respectively. The interface voltage and desolvation temperature were set to 2.0 kV and 300 °C, respectively. ^1^H-NMR and ^1^H–^1^H correlation spectroscopy (COSY) spectra of purified (all-*E*)-, (9*Z*)-, and (13*Z*)-astaxanthin were recorded using a Bruker Avance NEO 500 MHz NMR spectrometer equipped with a 5 mm iSmart Probe (Bruker Corp., Billerica, MA, USA) at a magnetic field strength of 11.7 T at 25 °C. For the NMR analysis, 5.0 mg of each astaxanthin isomer was dissolved in ∼0.5 mL of CDCl_3_.^27,29^

### 2.6. Pharmacokinetics (PK) analysis

Non-compartmental analysis (NCA) employed the linear-up/log-down trapezoidal rule to derive exposure metrics. The primary endpoint was AUC_0–∞_, computed as AUC_0–*t*_last__ + *C*_last_/*λ*_z_, where *t*_last_ and *C*_last_ denote the time and concentration at the last quantifiable sample. The terminal rate constant *λ*_z_ was estimated from the terminal log-linear phase by ordinary least-squares regression using the last 3–5 positive concentrations; among candidate windows, the fit with the highest *R*^2^ and *λ*_z_ > 0 was retained. Concentrations below the lower limit of quantification (BLQ) were treated as missing for NCA (no imputation). *C*_max_ and *T*_max_ were taken directly from observations; for summary statistics, *T*_max_ is reported as both the arithmetic mean and the median. Absolute oral bioavailability was estimated as *F*_NCA_ = (AUC_PO × Dose_IV)/(AUC_IV × Dose_PO), and intravenous systemic clearance was estimated as CL_NCA_ = Dose_IV/AUC_IV, where oral (per os, PO) and intravenous (IV) denote the respective routes of administration. The results are presented as mean ± standard deviation (SD), reflecting inter-individual variability. Structural models (one- to three-compartments ± absorption lag, transit chains, entero-hepatic circulation, and lipid pool) were coded in SciPy 1.11 sing least_squares^31^ and ranked by Akaike's information criterion (AIC). A more complex model was retained only when ΔAIC ≤ −2 and all additional parameters were identifiable (relative standard error <50%), in accordance with the information-theoretic framework proposed by Burnham and Anderson.^32^ The one-compartment + lag (1C + *T*_lag_) model met these criteria for all isomers. Uncertainty was quantified using 200 non-parametric bootstrap replicates stratified by the compound and administration route. The 2.5th and 97.5th percentiles of the bootstrap distribution defined the 95% confidence intervals (CIs) for each parameter. All analyses were performed in Python 3.11 with pandas 2.2, NumPy 1.26, and SciPy 1.11.^31^

### 2.7. Molecular docking simulation

Molecular docking simulations were conducted to investigate the binding affinities and interaction profiles of the astaxanthin isomers (the all-*E*-, 9*Z*-, and 13*Z*-isomers) and key proteins (*i.e.*, β-carotene oxygenase-2 [BCO2], scavenger receptor class B type I [SR-BI], cluster of differentiation 36 [CD36], serum albumin [SA], and apolipoprotein AI [apoA-I]) involved in the absorption, transport, metabolism, and systemic distribution of carotenoids.^18,33–37^ The crystal structures or predicted models of the following proteins were used in the docking study: BCO2 (AlphaFold Protein Structure Database, UniProt ID: Q99NF1), SR-BI (AlphaFold Protein Structure Database, UniProt ID: Q61009), CD36 (PDB ID: 5LGD), SA (PDB ID: 4F5S), and apoA-I (PDB ID: 1AV1). All protein structures were prepared using the structure preparation module in Molecular Operating Environment (MOE), including protonation and removal of the crystallographic water molecule. Prior to docking, ligand structures of each astaxanthin isomer were drawn and energy-minimized using MOE. The docking sites were defined based on known ligand-binding regions, particularly those reported or predicted to accommodate lipophilic compounds such as carotenoids. For BCO2 and SR-BI, the sites were set to be near the predicted carotenoid-binding regions, based on the binding sites proposed by Daruwalla and Kiser (2020)^33^ and Yang *et al.* (2016),^18^ respectively. For CD36, the site was set near the palmitic acid-binding region based on the co-crystallized ligand in the PDB structure 5LGD.^34^ For apoA-I, the docking site was set to be on the surface of the tetrameric structure in the PDB structure 1AV1, based on docking studies with cholesterol reported by Baserova *et al.* (2021).^36^ For SA, the docking site was defined in accordance with previous studies using PDB structure 4F5S, which has been utilized in docking simulations of astaxanthin isomers.^37^ Docking simulations were performed using the dock module implemented in MOE. Docking scores were calculated using the scoring functions available in the software, and the most favorable binding pose was selected based on the lowest energy score. Binding orientations and interacting amino acid residues were visualized using three-dimensional (3D) surface representations and two-dimensional (2D) interaction diagrams generated by the software.

### 2.8. Statistical analysis

Data are expressed as the mean ± standard error of the mean (SEM) from four mice per group. Statistical comparisons were performed using one-way ANOVA followed by Tukey's post-hoc test, using EZR software (version 1.54; Saitama Medical Center, Jichi Medical University, Saitama, Japan). Statistical significance was defined as *p* < 0.05.

## 3. Results and discussion

### 3.1. Identification of astaxanthin isomers

**Fig. 1:**
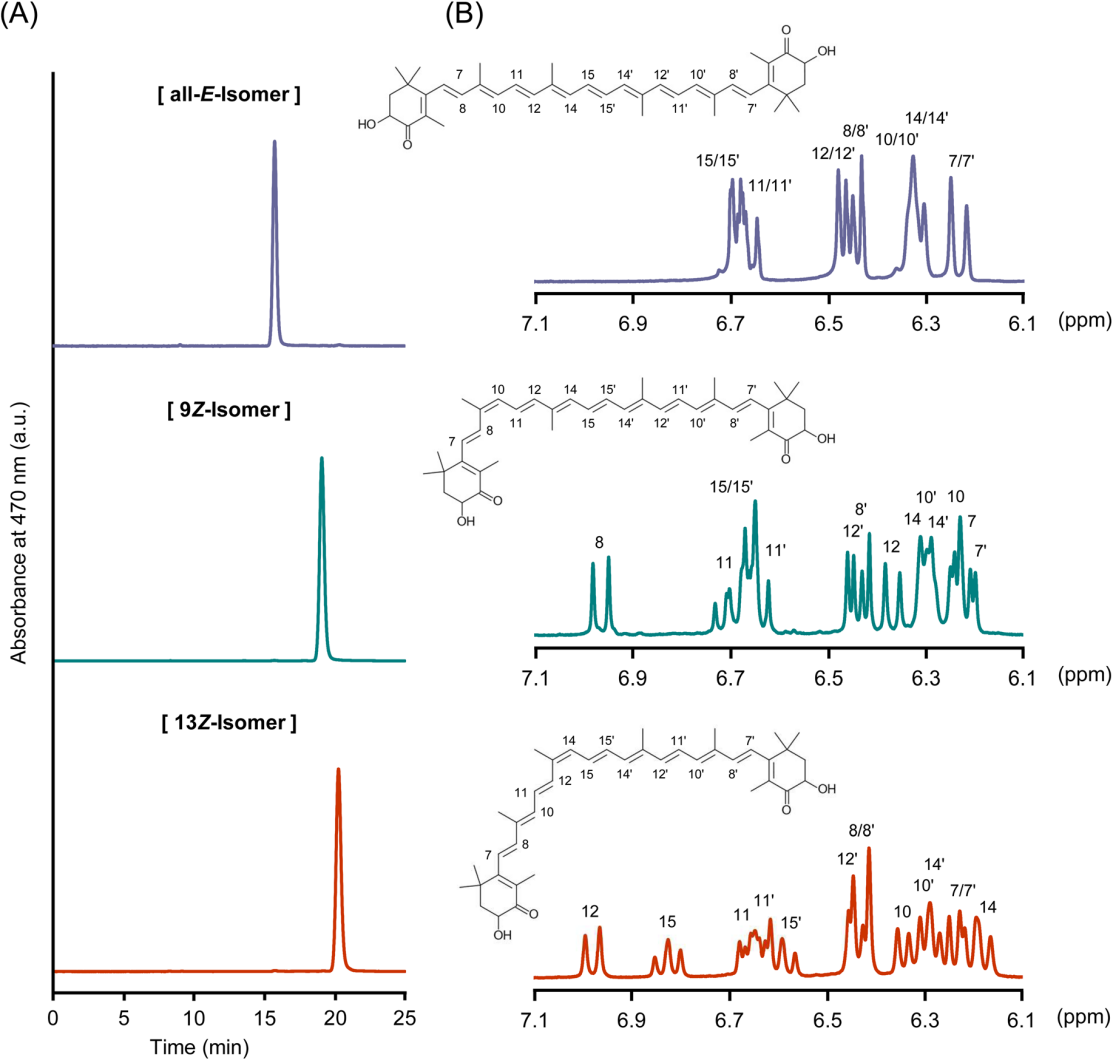
(A) Normal-phase HPLC chromatograms of purified (all-*E*)-, (9*Z*)-, and (13*Z*)-astaxanthin, and (B) their ^1^H-NMR spectra in CDCl_3_ in the range of 7.1–6.1 ppm, with peak assignments indicated.

Identification of astaxanthin isomers. The astaxanthin isomers were successfully isolated by scaling up the analytical method that we had recently developed (Fig. 1A).^20^ The purities of (all-*E*)-, (9*Z*)-, and (13*Z*)-astaxanthin, as determined by HPLC, were 98.3%, 99.2%, and 98.8%, respectively. In the mass spectrometry analysis, they were detected as quasimolecular ions at *m*/*z* 597.5 [M + H]^+^ (Fig. S1). The absorption maxima (*λ*_max_) of the (all-*E*)-, (9*Z*)-, and (13*Z*)-isomers in a solvent mixture of hexane, ethyl acetate, and acetone (75 : 23 : 2, v/v/v) were 472, 466, and 464 nm, respectively (Fig. S2). The 9*Z*- and 13*Z*-isomers exhibited *Z*-specific absorption bands (*Z*-peaks) around 365 nm, with *Q*-ratios, defined as the relative intensities of the *Z*-peak in relation to the principal absorption peak of the isomer, of 0.19 and 0.49, respectively (note that the *Z*-peak of the 9*Z*-isomer shows a shoulder-like shape). The characteristics of these absorption spectra were consistent with those reported in previous studies.^27,28^ Furthermore, the ^1^H-NMR and ^1^H–^1^H COSY spectra were consistent with previous reports (Fig. 1B and S3–S5).^29,30^ For example, in (9*Z*)-astaxanthin, the inward-facing protons, such as 8-H and 11-H, shifted to a lower field, and the outward-facing protons, such as 10-H and 12-H, shifted to a higher field compared to those of the all-*E*-isomer (Fig. 1B, S3, and S4). Similarly, in (13*Z*)-astaxanthin, the inward-facing protons, such as 12-H and 15-H, shifted to a lower field, and the outward-facing protons, such as 14-H and 15′-H, shifted to a higher field compared to those of the all-*E*-isomer (Fig. 1B, S3 and S5). These data not only support the assignment of the isomers but also ensure that the separated samples are of high purity. Numerous studies have investigated the differences in the bioavailability and pharmacokinetics of astaxanthin *E*/*Z*-isomers.^14–16,21,22^ However, these studies have typically used low-purity *Z*-isomer samples containing mixtures of multiple isomers. Accordingly, this study provides a more precise evaluation of the bioavailability and pharmacokinetics of astaxanthin using individually purified isomers.

### 3.2. Bioavailability and pharmacokinetics of astaxanthin isomers

#### 3.2.1. Plasma concentration behavior in oral administration (PO) study

**Fig. 2:**
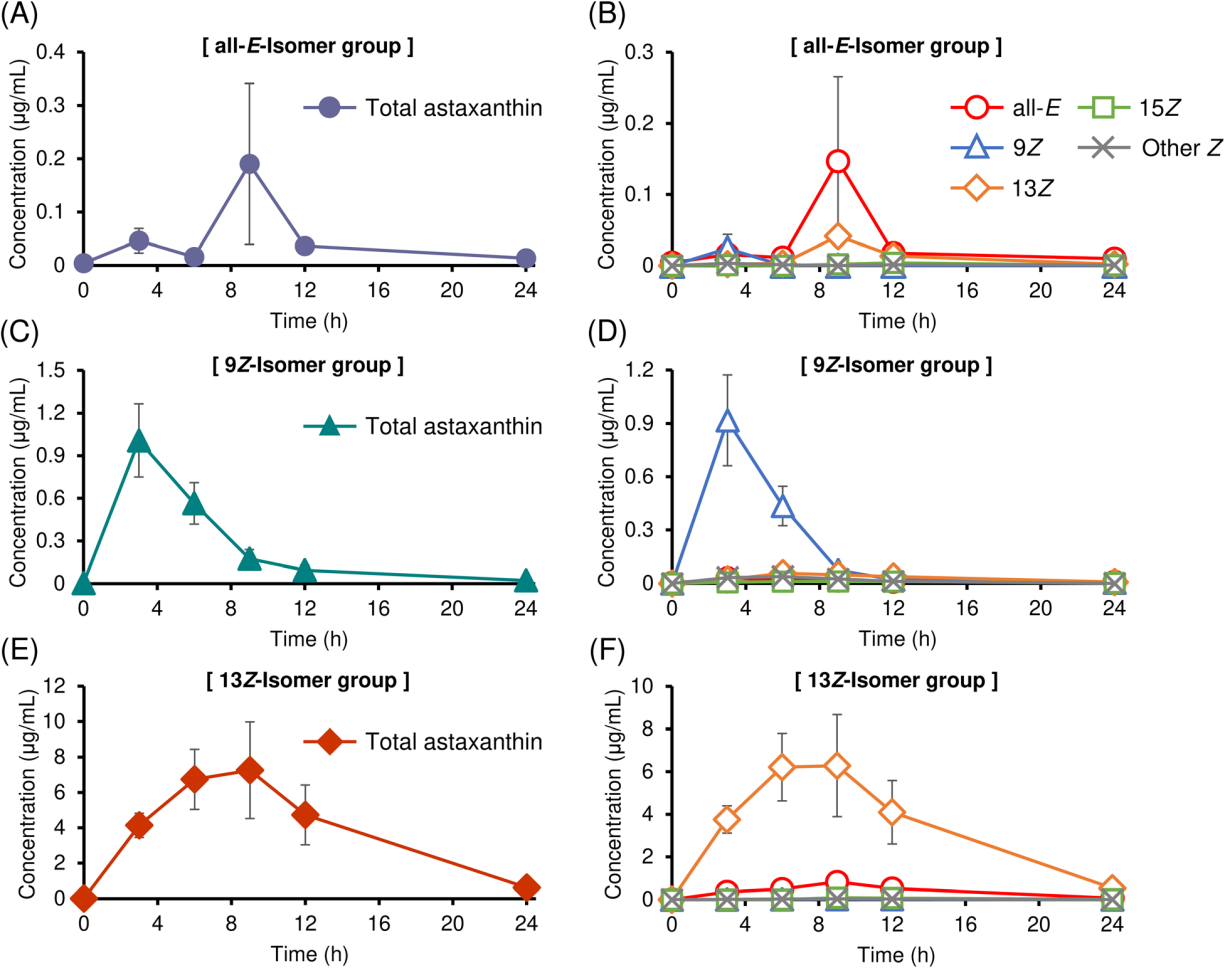
Time profiles of (A, C and E) total astaxanthin concentration (μg mL^−1^) and (B, D and F) each astaxanthin isomer concentration (μg mL^−1^) in plasma after oral (PO) administration of (A and B) (all-*E*)-, (C and D) (9*Z*)-, and (E and F) (13*Z*)-astaxanthin. Labels of (all-*E*), (9*Z*), (13*Z*), and (15*Z*) denote (all-*E*)-, (9*Z*)-, (13*Z*)-, and (15*Z*)-astaxanthin, respectively, and (other *Z*) denotes the sum of unknown astaxanthin *Z*-isomers (perhaps multi-*Z*-isomers). Data are presented as mean ± SEM (*n* = 4).

Time profiles of astaxanthin isomer concentrations in plasma samples after oral administration of purified astaxanthin isomers dispersed in soybean oil (100 mg kg^−1^) to mice are shown in Fig. 2A, C, and E. All astaxanthin treatments led to an increase in plasma astaxanthin concentrations; however, the extent and pattern of increase varied markedly depending on the isomer administered. Specifically, plasma concentrations followed the order of the 13*Z*- > 9*Z*- > all-*E*-isomers. The plasma concentrations of astaxanthin at the peak time point following administration of the all-*E*-, 9*Z*-, and 13*Z*-isomers were 0.19 ± 0.15 μg mL^−1^, 1.01 ± 0.51 μg mL^−1^, and 7.70 ± 5.09 μg mL^−1^, respectively. The plasma concentration profiles in the all-*E* isomers group showed slight biphasic peak. Although several studies have reported that administration of astaxanthin *Z*-isomers leads to higher plasma astaxanthin concentrations compared to the all-*E*-isomer,^14–16,21,22^ these studies employed mixtures of various *Z*-isomers, and thus did not elucidate the differences in absorbability among individual *Z*-isomers. The present study is the first to demonstrate that absorbability differs significantly among individual *Z*-isomers. The time-dependent plasma concentration profiles of each isomer suggest the possibility of *in vivo* isomerization of astaxanthin during systemic circulation (Fig. 2B, D and F). This aspect is discussed in detail in Section 3.3.1. In addition to differences in peak plasma concentrations depending on the isomer, the time-course profiles of astaxanthin levels also varied significantly among the isomers (Fig. 2A, C and E). Notably, the 9*Z*-isomer reached its peak concentration earlier and declined more rapidly than the all-*E*- and 13*Z*-isomers. The plasma concentration of the 13*Z*-isomer gradually increased following oral administration, reaching a delayed peak and remaining elevated for a longer duration compared to the other isomers. This suggests that the 13*Z*-isomer may be retained in systemic circulation for an extended period. A similar trend has also been observed in a previous study involving oral administration of mixed astaxanthin isomers to rats.^38^ Detailed pharmacokinetic analysis, including intravenous administration, is presented in Section 3.2.3.

#### 3.2.2. Plasma concentration behavior in bolus intravenous injection (IV) study

**Fig. 3:**
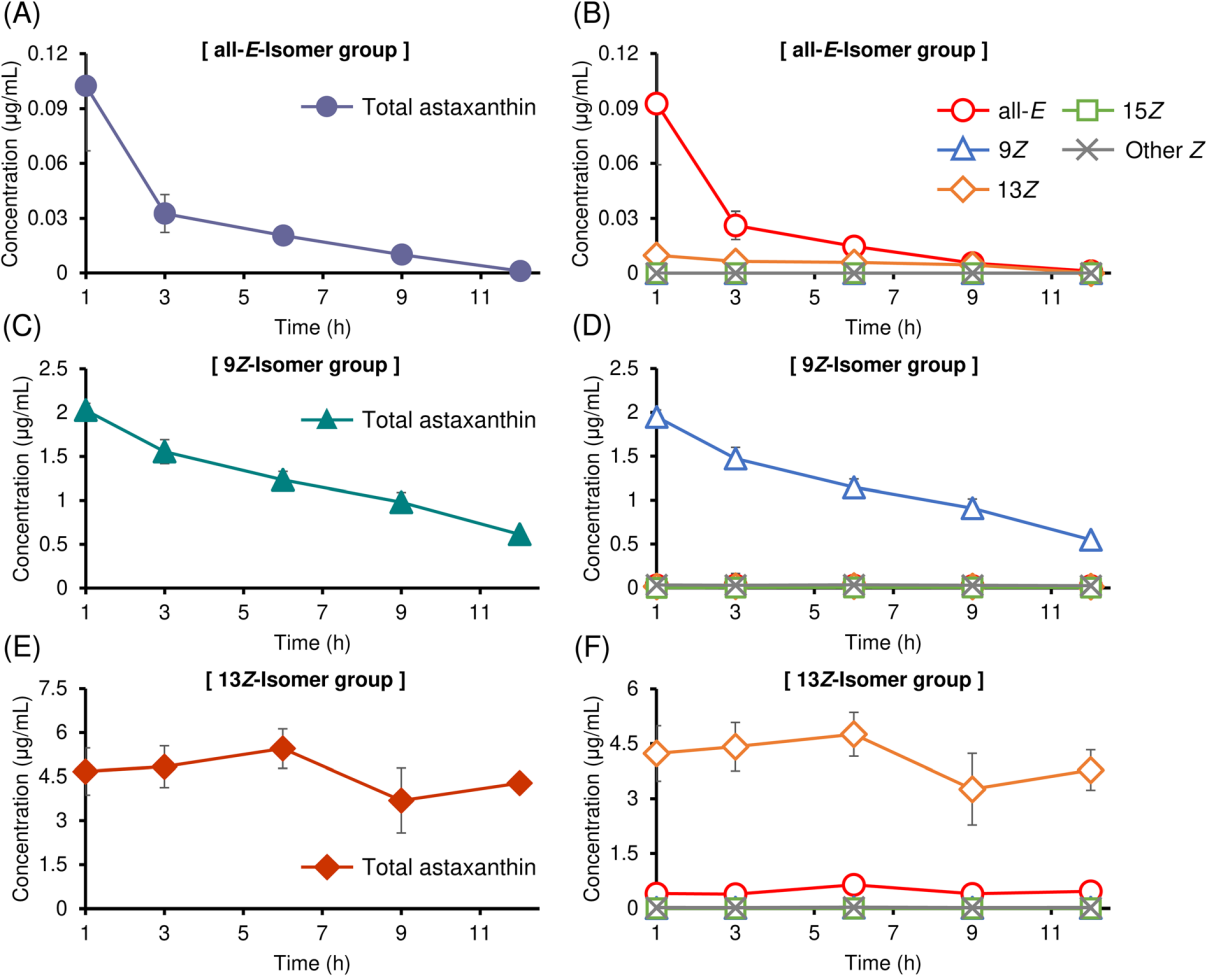
Time profiles of (A, C and E) total astaxanthin concentration (μg mL^−1^) and (B, D and F) each astaxanthin isomer concentration (μg mL^−1^) in plasma after intravenous (IV) administration of (A and B) (all-*E*)-, (C and D) (9*Z*)-, and (E and F) (13*Z*)-astaxanthin. Labels of (all-*E*), (9*Z*), (13*Z*), and (15*Z*) denote (all-*E*)-, (9*Z*)-, (13*Z*)-, and (15*Z*)-astaxanthin, respectively, and (other *Z*) denotes the sum of unknown astaxanthin *Z*-isomers (perhaps multi-*Z*-isomers). Data are presented as mean ± SEM (*n* = 4).

Time profiles of astaxanthin isomer concentrations in plasma samples after bolus intravenous administration of purified astaxanthin isomers dispersed in a mixture of *N*,*N*-dimethylacetamide and polyethylene glycol 400 (1 : 1, v/v) (10 mg kg^−1^) in mice are shown in Fig. 3A, C, and E. All astaxanthin samples showed an increase in plasma astaxanthin concentration; however, the concentration varied greatly depending on the type of isomer administered. The trend was similar to that observed in the oral administration study; that is, the plasma astaxanthin concentration was highest in the 13*Z*-isomer administration group, followed by the 9*Z*- and all-*E*-isomer administration groups. The observed differences in plasma concentrations are likely attributable to varying incorporation efficiencies of the astaxanthin isomers into circulating lipoproteins (*e.g.*, chylomicrons and very-low-density lipoproteins [VLDLs]), which mediate the systemic distribution of carotenoids.^35^ Therefore, the higher plasma appearance of the 13*Z*-isomer observed in the PO study may be, at least in part, attributed to its higher affinity for plasma lipoproteins. Similar to the PO study, the time-dependent plasma concentration profiles of each isomer suggest the possibility of *in vivo* isomerization of astaxanthin during systemic circulation (Fig. 2B, D and F). This aspect is discussed in detail in Section 3.3.2. Following intravenous administration of the all-*E*- and 9*Z*-isomers, plasma astaxanthin concentrations gradually decreased over time (Fig. 3A and C). In contrast, after administration of the 13*Z*-isomer, a slight decrease over time was observed, but the concentration remained mostly constant for up to 12 h (Fig. 3E). This observation indicates that the 13*Z*-isomer is more stable and less susceptible to metabolic degradation and elimination from mice bodies than the all-*E*- and 9*Z*-isomers. Alternatively, the persistence of the 13*Z*-isomer in the plasma may indicate its participation in enterohepatic circulation.^39^ Detailed pharmacokinetic analysis, including PO administration, is presented in Section 3.2.3.

#### 3.2.3. Pharmacokinetic (PK) analysis

To characterize pharmacokinetic (PK) differences among astaxanthin isomers, we first estimated clearance (CL) and absolute bioavailability (*F*) by non-compartmental analysis (NCA) after oral (100 mg kg^−1^) and intravenous (10 mg kg^−1^) dosing, assuming dose-proportional exposure.^31,32^ NCA revealed clear isomer-specific profiles (Table 1): the 13*Z*-isomer showed lower CL with sustained exposure; the 9*Z*-isomer showed higher CL indicative of faster elimination; and the all-*E*-isomer exhibited very low exposure, consistent with rapid systemic loss. Oral *F* was uniformly low (approximately 4–18%) across isomers, in line with the formulation-dependent low bioavailability of astaxanthin.^40,41^ To investigate mechanisms, we evaluated structural models (one to three compartments) incorporating absorption lag time (*T*_lag_), enterohepatic recirculation (EHC), and a lipid-pool component, and ranked candidates by Akaike's Information Criterion (AIC). Across compounds, the one-compartment + *T*_lag_ (1C + *T*_lag_) model provided the best balance of fit and parsimony (lowest AIC). Although the all-*E*-isomer profiles appeared biphasic, more complex structures (*e.g.*, 2C + *T*_lag_ or 1C + *T*_lag_ + lipid pool) neither improved fit meaningfully (ΔAIC ≥ +1.2) nor met identifiability criteria (relative standard error >50%; Table S1). Final parameter estimates and 95% confidence intervals were obtained *via* a 200-replicate non-parametric bootstrap stratified by compound and route (Table 2). The observed ranking is mechanistically plausible given biological interactions and physicochemical properties. Differences in enzymatic selectivity, crystallinity, solubility, lipoprotein affinity, and plasma-protein binding likely contribute. In particular, β-carotene oxygenase-2 (BCO2) preferentially cleaves *Z*-isomers at the C_9,10_ double bond; for example, chicken BCO2 shows much higher catalytic efficiency toward (9*Z*)-β-carotene than toward the all-*E* isomer, consistent with enhanced first-pass/rapid metabolism of the 9*Z*-isomer.^33,42^ As detailed later (Section 3.4), our docking analysis indicated stronger BCO2 binding for the 9*Z*-isomer than for the 13*Z*-isomer, in line with the higher CL observed for the 9*Z*-isomer in mice. Conversely, the 13*Z*-isomer appears to benefit from improved bioaccessibility (reduced crystallinity → faster dissolution/micellarization) and less pronounced metabolic loss, supporting its higher systemic and hepatic levels *versus* the 9*Z*-isomer. Although the *Z*-isomers generally display better bioaccessibility than the all-*E*-isomer, the net exposure of the 9*Z*-isomer remains limited, plausibly due to its greater susceptibility to enzymatic clearance in the liver and/or intestine.^8,43^ These findings agree with prior animal studies (rats, guinea pigs, rainbow trout) in which diets containing both the 9*Z*- and 13*Z*-isomers yielded low 9*Z*- and predominant 13*Z*-isomer levels in plasma and tissues.^14,15,22^ They may also help reconcile long-standing discrepancies in β-carotene bioavailability: the all-*E*-isomer-rich preparations often produce higher plasma β-carotene than natural 9*Z*-isomer-rich sources (*e.g.*, *Dunaliella* sp.), whereas the 13*Z*-isomer-rich (heat-isomerized) preparations can exceed the all-*E*-isomer in bioavailability.^44–48^

**Table 1:** Non-compartmental pharmacokinetic parameters of astaxanthin isomers^a^

	Route^b^	AUC_0–∞_ (mg h L^−1^)	*C*_max_ (mg L^−1^)	*T*_max_ c (h)	CL_NCA_ (L h^−1^ kg^−1^)	*F*_NCA_
(all-*E*)-Astaxanthin	PO	1.46 ± 1.35	0.01 ± 0.01	3 (9)^d^	—	0.12
	IV	0.31 ± 0.20	0.10 ± 0.07	—	35.71	—
(9*Z*)-Astaxanthin	PO	4.33 ± 2.07	1.01 ± 0.51	4	—	0.04
	IV	19.40 ± 3.30	2.03 ± 0.16	—	0.74	—
(13*Z*)-Astaxanthin	PO	82.20 ± 57.40	7.70 ± 5.09	6	—	0.18
	IV	127.10 ± 32.40	5.46 ± 1.34	—	0.19	—

a Each value is presented as mean ± standard deviation (SD), derived from individual animal profiles (*n* = 4 per group).

b Oral (per os, PO) or intravenous (IV) routes.

c *T*_max_ for each sample is shown as the median.

d Some of animals showed biphasic peaks.

**Table 2:** Population pharmacokinetic parameter estimates for astaxanthin isomers using a 1-compartment + absorption lag (1C + *T*_lag_) model^a^

	CL (L h^−1^ kg^−1^)	*V* (L kg^−1^)	*k*_a_ (h^−1^)	*F*	*T*_lag_ (h)
(all-*E*)-Astaxanthin	27 (18–40)	75 (39–122)	52 (4–210)	0.12 (0.04–0.30)	0.66 (0.25–1.4)
(9*Z*)-Astaxanthin	0.45 (0.40–0.51)	4.6 (4.1–5.2)	0.57 (0.08–3.1)	0.06 (0.03–0.10)	2.4 (1.0–3.7)
(13*Z*)-Astaxanthin	0.033 (0.021–0.049)	1.9 (1.5–2.3)	0.17 (0.05–0.62)	0.21 (0.10–0.36)	0.12 (0.04–0.28)

a Values are reported as median (95% confidence interval) based on 200-fold non-parametric bootstrap stratified by compound and route. Model parameters include: *k*_a_ (absorption rate constant, h^−1^), *T*_lag_ (absorption lag time, h), CL (systemic clearance, L h^−1^ kg^−1^), *V*_d_ (apparent volume of distribution, L kg^−1^). This model selection was based on Akaike's Information Criterion (AIC); the 1C + *T*_lag_ model was preferred across all isomers (see Table S1).

Taken together with Table 2, the 1C + *T*_lag_ PopPK estimates provide a coherent, system-level explanation for the isomer-specific exposure patterns observed in the NCA. (13*Z*)-Astaxanthin combines a shorter absorption *T*_lag_ with lower CL and a moderate *k*_a_, yielding sustained systemic exposure after PO dosing and aligning with its higher hepatic levels. In contrast, (9*Z*)-astaxanthin shows higher CL and a longer *T*_lag_ despite a relatively faster *k*_a_, which translates into rapid elimination with a delayed onset and therefore limited oral exposure. For the all-*E*-isomer, the model required larger V and CL to reconcile the profiles, consistent with weak plasma retention and the biphasic appearance noted in the concentration–time curves; the wider confidence intervals suggest potential distributional heterogeneity that could be explored with richer sampling or multi-compartment extensions. Importantly, these parameters bridge molecular interaction tendencies and *in vivo* disposition: the stronger ApoA-I/serum-albumin binding inferred for the 13*Z*-isomer supports prolonged circulation, whereas a plausible BCO2-mediated metabolic route for the 9*Z*-isomer is consistent with faster removal (see Table 3). Overall, Table 2 adds explanatory power beyond summary PK by integrating route effects (*F*), onset kinetics (*T*_lag_), and elimination (CL), thereby rationalizing why the 13*Z*-isomer achieves higher systemic and hepatic exposure, while the 9*Z*-isomer remains low after PO administration.

For robustness, we note that median (95% CI) parameter estimates in Table 2 were consistent across bootstrap replicates stratified by compound and route, and the preferred 1C + *T*_lag_ structure met the pre-specified identifiability and AIC criteria. While IV sampling ended at 12 h, exposure comparisons in the main text are now based on AUC_0–∞_ to avoid ambiguity from extrapolation. Finally, because several PO profiles exhibit low concentrations and occasional biphasic shapes, conclusions for the all-*E*-isomer are interpreted with appropriate caution; targeted future designs (denser early PO sampling and extended IV sampling) would further refine *T*_lag_, *k*_a_, and distributional parameters.

**Table 3:** Docking scores (kcal mol^−1^) between proteins and astaxanthin isomers^a^

	SR-B1	CD36	ApoA-I	SA	BCO2
(all-*E*)-Astaxanthin	−8.59	−7.09	−7.42	−6.98	−10.26
(9*Z*)-Astaxanthin	−9.15	−9.27	−8.40	−7.74	−10.77
(13*Z*)-Astaxanthin	−9.07	−9.96	−8.55	−8.44	−9.62

a More negative values indicate stronger predicted binding affinities.

### 3.3. *In vivo* isomerization behavior of astaxanthin isomers

#### 3.3.1. Isomerization behavior in oral administration (PO) study

**Fig. 4:**
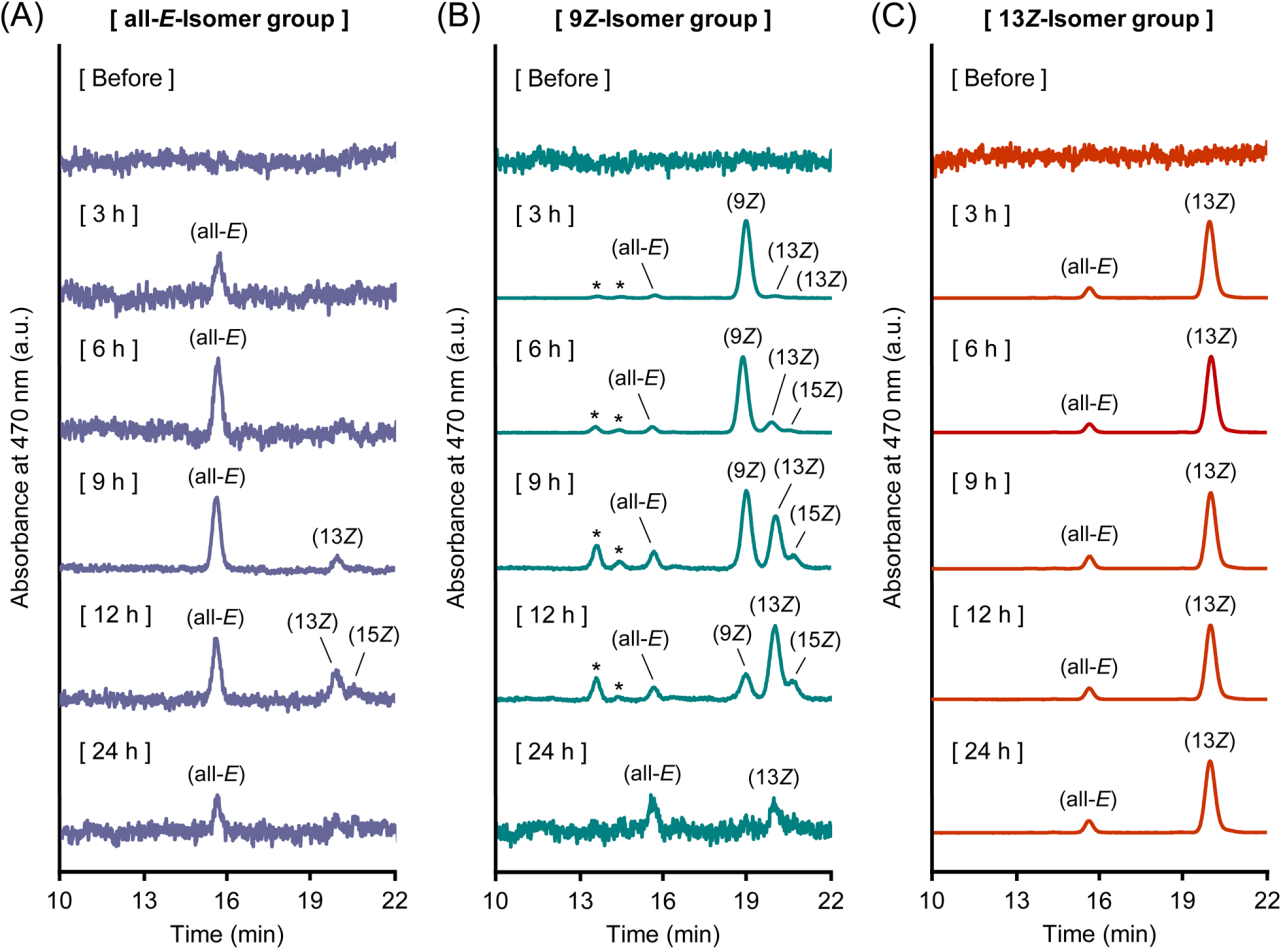
(A) Normal-phase HPLC chromatograms of plasma samples after oral administration of (A) (all-*E*)-, (B) (9*Z*)-, and (C) (13*Z*)-astaxanthin. Labels of (all-*E*), (9*Z*), (13*Z*), and (15*Z*) denote (all-*E*)-, (9*Z*)-, (13*Z*)-, and (15*Z*)-astaxanthin, respectively. The peaks marked with an asterisk (*) are tentatively identified as astaxanthin *Z*-isomers according to the spectrum data.^26–28^

Chromatographic analysis and time-course plasma profiles of each isomer clearly demonstrated that astaxanthin isomers isomerize to other forms during absorption or systemic circulation (Fig. 2B, D, F and 4). After oral administration, a small portion of (all-*E*)-astaxanthin was converted to the 13*Z*- and 15*Z*-isomers (Fig. 2B and 4A). By comparison, (9*Z*)-astaxanthin isomerized into a variety of *Z*-isomers, including the all-*E*-, 13*Z*-, and 15*Z*-isomers, and two unidentified *Z*-isomers (Fig. 2D and 4B). In addition, a minor portion of the 13*Z*-isomer isomerized to the all-*E*-isomer (Fig. 2F and 4C). This indicates that (9*Z*)-astaxanthin may be more prone to isomerize (metabolize) into other isomeric forms compared to the all-*E*- and 13*Z*-isomers in the body. Interestingly, although the 13*Z*-isomer is reported to have lower thermodynamic stability and to be less stable than the all-*E*- and 9*Z*-isomers, making it prone to isomerize into the more stable all-*E*-isomer,^49,50^ it was found to exist stably in the bodies of mice. Specifically, in plasma from the 13*Z*-isomer administration group, no time-dependent increase in the all-*E*-isomer was observed, and the 13*Z*-isomer remained in the predominant form throughout the 24 h period. Although the stabilization mechanism was not directly examined in this study, carotenoids are known to be incorporated into plasma lipoproteins and can also associate with serum albumin.^35^ Thus, carrier interactions could plausibly contribute to the *in vivo* persistence of the 13*Z*-isomer, which is consistent with previous reports of enhanced stability of the *Z*-isomers through complex formation with macromolecules.^51,52^ Nevertheless, the exact mechanism remains to be clarified.

**Fig. 5:**
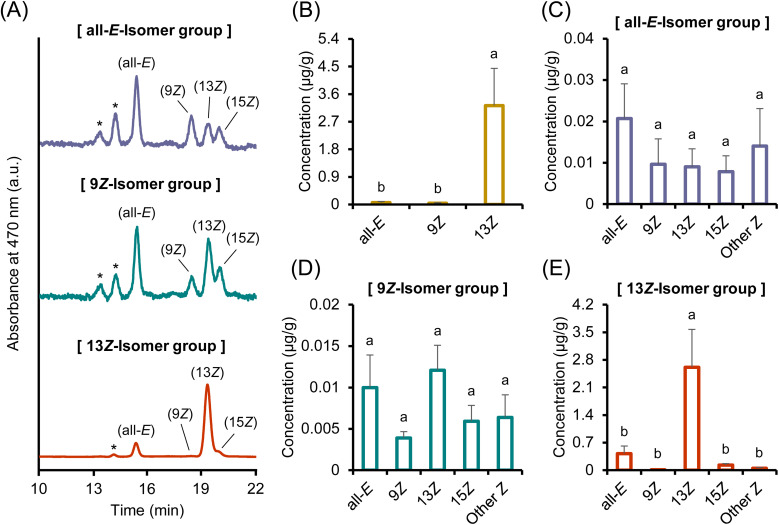
(A) Normal-phase HPLC chromatograms of liver samples after oral administration of astaxanthin isomers and (B) total astaxanthin concentration (μg mL^−1^) and (C, D and E) each astaxanthin isomer concentration (μg g^−1^) in liver samples after oral administration of (C) (all-*E*)-, (D) (9*Z*)-, and (E) (13*Z*)-astaxanthin. Labels of (all-*E*), (9*Z*), (13*Z*), and (15*Z*) in (A) denote (all-*E*)-, (9*Z*)-, (13*Z*)-, and (15*Z*)-astaxanthin, respectively. The peaks marked with an asterisk (*) are tentatively identified as astaxanthin *Z*-isomers according to the spectrum data.^26–28^ The means with different letters for each isomer in (B–E) are significantly different (*p* < 0.05). Data are presented as mean ± SEM (*n* = 4).

Fig. 5 shows the chromatograms and concentrations of astaxanthin isomers in the liver, collected 24 h after administration of the three different astaxanthin isomers. When the all-*E*- or 9*Z*-isomer was administered, isomers different from the administered form were abundantly detected in the livers of both groups. These isomeric changes may have occurred either before hepatic accumulation or in the liver. Notably, in the group administered the 9*Z*-isomer, its proportion in the liver was considerably lower than that of the other isomers, which may indicate that the 9*Z*-isomer was rapidly isomerized or metabolized. In contrast, when the 13*Z*-isomer was administered, it retained its isomeric form in the liver, with only a small amount of the all-*E*-isomer being detected, which was consistent with the plasma results. Carotenoids are known to undergo metabolic conversion in the liver through various enzymatic reactions.^53,54^ During this metabolic process, the all-*E*- and 9*Z*-isomers are possibly converted into other isomeric forms. Indeed, several reports have indicated that the proportion of carotenoid *Z*-isomers tends to be higher in the liver than in other tissues.^14,22,47^ Alternatively, Huang and Hui (2020) demonstrated that carotenoid lycopene may be converted to the *Z*-isomers during absorption across the small intestinal wall.^55^ Because orally administered carotenoids are absorbed through the small intestine into the bloodstream and subsequently transported to the liver for storage, an increase in the *Z*-isomerization in the small intestine wall may have contributed to the higher proportion of carotenoid *Z*-isomers observed in the liver. As an additional note, *in vitro* studies have shown that carotenoids minimally isomerize during gastric transit and the process of transferring to micelles.^56,57^ Consistently, in the present study, the administered all-*E*- or 9*Z*-isomer mainly retained its original isomeric structure in fecal samples (Fig. S6A, C and D). On the other hand, since the 13*Z*-isomer was found to persist stably in both plasma and liver, it may be less susceptible to metabolic conversion in the small intestine or liver. Incidentally, in the fecal samples obtained after administration of the 13*Z*-isomer, a large amount of the all-*E*-isomer was detected (Fig. S6A and E). This finding may suggest that although the 13*Z*-isomer remains stable *in vivo*, it is relatively unstable under *ex vivo* conditions, potentially undergoing isomerization into the thermodynamically more stable all-*E*-form after excretion.^49,50^ Furthermore, the astaxanthin concentration in the liver after the administration of the 13*Z*-isomer was approximately 52.7- and 84.3-fold higher than that observed after the administration of the all-*E*- and 9*Z*-isomers, respectively (Fig. 5B). Therefore, the administration of the 13*Z*-isomer was effective in enhancing astaxanthin levels in tissues and organs.

#### 3.3.2. Isomerization behavior in intravenous injection (IV) study

**Fig. 6:**
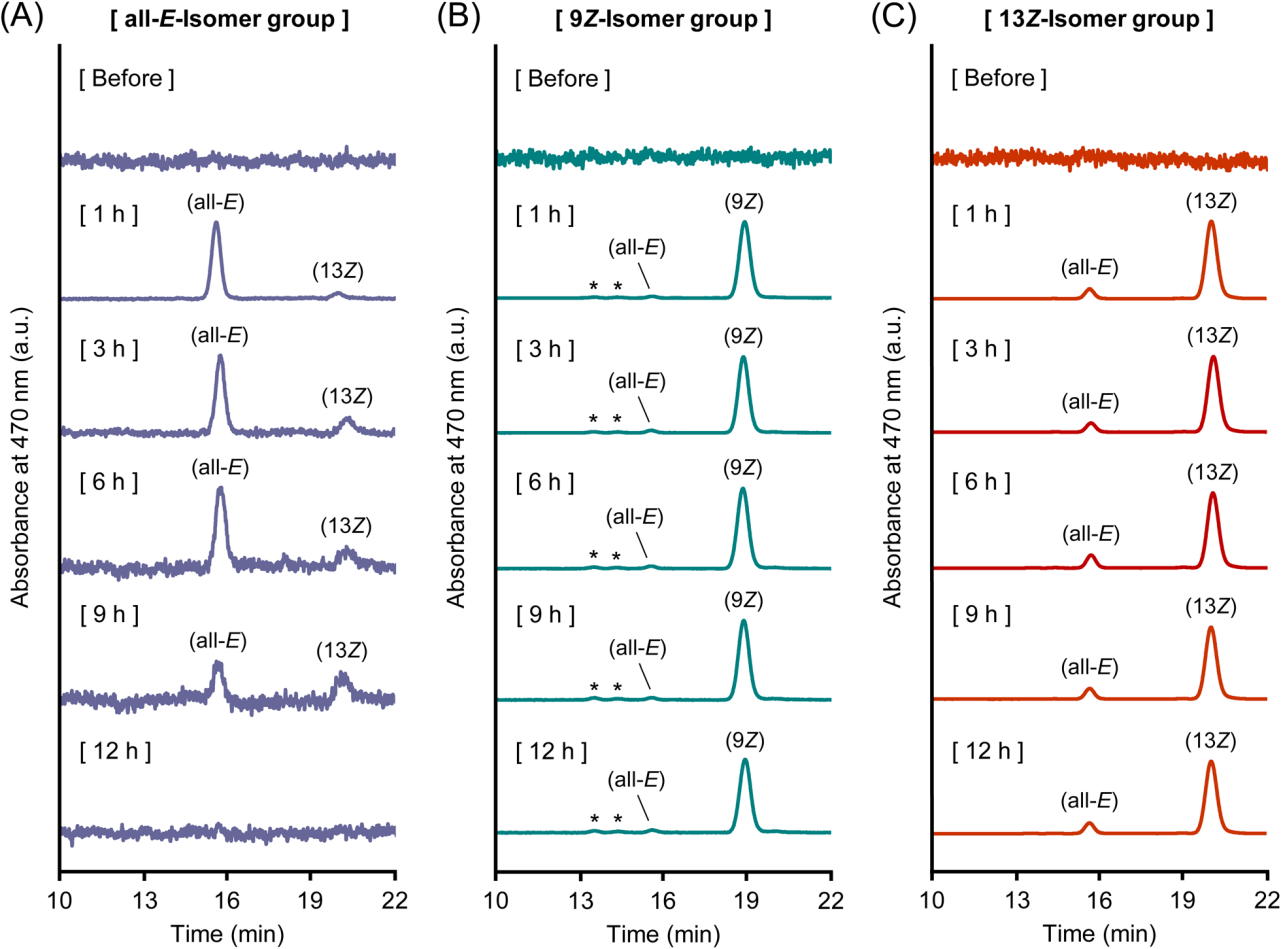
(A) Normal-phase HPLC chromatograms of plasma samples after intravenous administration of (A) (all-*E*)-, (B) (9*Z*)-, and (C) (13*Z*)-astaxanthin. Labels of (all-*E*), (9*Z*), (13*Z*), and (15*Z*) denote (all-*E*)-, (9*Z*)-, (13*Z*)-, and (15*Z*)-astaxanthin, respectively. The peaks marked with an asterisk (*) are tentatively identified as astaxanthin *Z*-isomers according to the spectrum data.^26–28^

The time-dependent isomerization patterns observed after intravenous administration of the all-*E*- and 13*Z*-isomers were consistent with those observed in the oral administration study (Fig. 3B, D, F and 6). In the all-*E*-isomer administration group, the proportion of the 13*Z*-isomer gradually increased over time (Fig. 3B and 6A). In the 13*Z*-isomer administration group, the isomer composition remained relatively stable, with the 13*Z*-isomer dominating throughout the observation period and only a small amount of the all-*E*-isomer was detected (Fig. 3F and 6C). In contrast, the 9*Z*-isomer showed markedly limited isomerization; only trace amounts of other isomers were detected, and the 9*Z*-isomer remained the dominant form throughout the observation period (Fig. 3D and 6B). This indicates that the 9*Z*-isomer was relatively resistant to *in vivo* isomerization under the conditions tested, in contrast to the dynamic interconversion observed in the oral administration study.

**Fig. 7:**
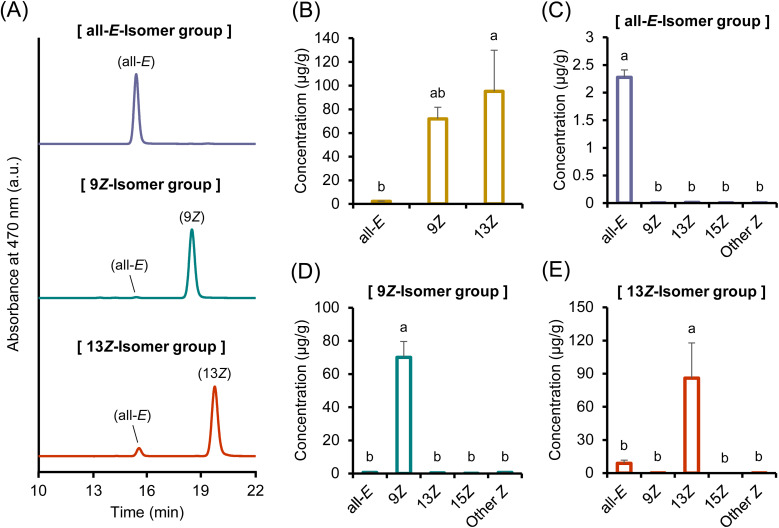
(A) Normal-phase HPLC chromatograms of liver samples after intravenous administration of astaxanthin isomers and (B) total astaxanthin concentration (μg mL^−1^) and (C–E) each astaxanthin isomer concentration (μg g^−1^) in liver samples after intravenous administration of (C) (all-*E*)-, (D) (9*Z*)-, and (E) (13*Z*)-astaxanthin. Labels of (all-*E*), (9*Z*), (13*Z*), and (15*Z*) in (A) denote (all-*E*)-, (9*Z*)-, (13*Z*)-, and (15*Z*)-astaxanthin, respectively. The means with different letters for each isomer in (B–E) are significantly different (*p* < 0.05). Data are presented as mean ± SEM (*n* = 4).

Furthermore, astaxanthin extracted from the liver 12 h after intravenous administration mainly retained the isomeric form that had been administered (Fig. 7). This finding contrasts markedly with the results of the oral administration study, in which notable isomerization occurred following the administration of the all-*E*- and 9*Z*-isomers (Fig. 5A, C and D). These observations indicate that astaxanthin does not readily isomerize in the liver. Given that significant isomerization of the 9*Z*-isomer was observed after oral but not intravenous administration, and only minimal isomerization was detected in fecal samples, the small intestinal wall may contribute to isomeric transformation. This interpretation is consistent with previous reports on lycopene isomerization during epithelial uptake.^55^ However, the precise site and mechanisms of isomerization remain to be clarified. Notably, the hepatic accumulation of astaxanthin was substantially higher following intravenous administration of the 9*Z*- and 13*Z*-isomers than that of the all-*E*-isomer (Fig. 7B), demonstrating that the astaxanthin *Z*-isomers had better tissue retention than the all-*E*-isomer. Although hepatic accumulation of the 9*Z*-isomer was minimal after oral administration (Fig. 5B), it was comparable to that of the 13*Z*-isomer after intravenous injection. These findings indicate that intravenous administration is more effective than oral administration in increasing the systemic availability of the 9*Z*-isomer. In other words, the markedly lower accumulation of the 9*Z*-isomer in the liver after oral administration may be attributed to its poor bioavailability or metabolic conversion during the absorption process. To clarify the differences in isomerization behavior and accumulation of astaxanthin isomers, it is essential to identify the specific metabolic enzymes responsible for carotenoid isomer metabolism and investigate the resulting metabolites. Addressing these issues is an important task for future studies.

### 3.4. Molecular docking simulation

To gain molecular insights into the differential interaction of astaxanthin geometric isomers with transport-related proteins, molecular docking simulations were conducted using (all-*E*)-, (9*Z*)-, and (13*Z*)-astaxanthin and four key proteins involved in the absorption and transport of carotenoids: SR-BI, CD36, ApoA-I, and SA.^18,33–37^ SR-BI is known to play a key role in the absorption of dietary lipids by mediating the selective uptake of hydrophobic molecules.^18^ CD36 is a high-affinity scavenger receptor that is broadly expressed in various tissues and facilitates the cellular uptake of hydrophobic molecules.^34^ ApoA-I is the major protein component of HDL particles and is responsible for transporting hydrophobic molecules in the plasma.^35,36^ SA functions as a non-specific carrier of hydrophobic molecules in systemic circulation.^37^ In addition to these four transport-related proteins, BCO2, a carotenoid-cleaving enzyme involved in metabolic degradation rather than transport, was also included in the simulation.^33,42^ While BCO2 does not function as a transport-related protein, it plays a crucial role in regulating the systemic fate of carotenoids by catalyzing oxidative cleavage.

**Fig. 8:**
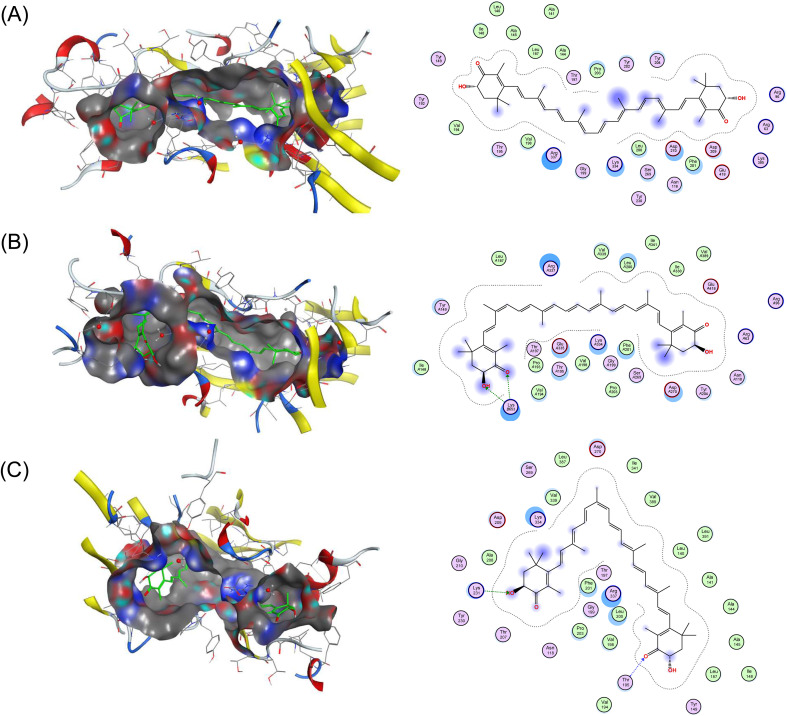
The 3D (binding pocket surface representation) and 2D interaction diagrams of CD36 with (A) (all-*E*)-, (B) (9*Z*)-, and (C) (13*Z*)-astaxanthin generated using MOE software. The 3D structures illustrate the ligand binding poses within the surface contour of the predicted binding pocket of CD36, while the 2D diagrams show the detailed molecular interactions with surrounding amino acid residues.

Docking simulations revealed that astaxanthin *Z*-isomers, particularly the 13*Z*-isomer, exhibited more favorable binding affinities across all transport-related target proteins than the all-*E*-isomer (Table 3). Among the four proteins, the 13*Z*-isomer demonstrated the strongest interaction energies with CD36 (−9.96 kcal mol^−1^), ApoA-I (−8.55 kcal mol^−1^), and SA (−8.44 kcal mol^−1^). In the case of SR-BI, although the 9*Z*-isomer showed the highest score (−9.15 kcal mol^−1^), the 13*Z*-isomer (−9.07 kcal mol^−1^) also outperformed the all-*E*-isomer (−8.59 kcal mol^−1^). These results indicate that the *Z*-isomerization increases the affinity of astaxanthin for absorption- and transport-related proteins compared. This trend was further supported by the structural analysis of the docking poses. The bent geometry of the *Z*-isomers allowed for a more compact fit within the hydrophobic binding pockets of each protein, resulting in a larger number of stabilizing interactions (Fig. 8 and S7–S9). This consistency is further supported by previous experimental and computational findings.^18,34,37^ For example, Zheng *et al.* (2024) reported that (13*Z*)-astaxanthin exhibited the highest binding affinity to SA among the all-*E*, 9*Z*, and 13*Z*-isomers, as demonstrated by both fluorescence spectroscopy and molecular docking simulations.^37^ Furthermore, the amino acid residues located in proximity to the astaxanthin-binding pocket of SA (*e.g.*, Leu189, Glu424, and Arg435) were consistent with those identified in the same study (Fig. S9). In contrast, docking scores for BCO2 revealed a distinct trend. The 9*Z*-isomer exhibited the strongest binding affinity to BCO2 (−10.77 kcal mol^−1^), followed by the all-*E*-isomer (−10.26 kcal mol^−1^) and the 13*Z*-isomer (−9.62 kcal mol^−1^) (Table 3 and Fig. S10). This result is consistent with the previous enzymatic study indicating that BCO2 preferentially cleaves 9*Z*-configured carotenoids.^42^ As discussed in Section 3.2.3, this may partially explain the rapid clearance and limited systemic retention of the 9*Z*-isomer observed *in vivo*. Conversely, the weaker affinity of the 13*Z*-isomer for BCO2 may contribute to its higher metabolic stability and prolonged circulation. Furthermore, the present study suggests that the 9*Z*-isomer may be metabolized more extensively in the small intestine than in the liver. Recent evidence indicates that BCO2 is strongly expressed in the small intestinal epithelium, where it contributes to the initial metabolism of dietary carotenoids.^58^ Taken together with our docking results showing preferential binding of the 9*Z*-isomer to BCO2, this raises the possibility that intestinal BCO2 substantially contributes to its first-pass metabolism and reduced systemic availability.

The results of the current docking simulations offer a mechanistic rationale for the enhanced oral absorption and prolonged systemic retention of the *Z*-isomers, as observed under both oral and intravenous administration conditions in this study (Fig. 2 and 3). The strong binding of the 13*Z*-isomer to circulating proteins such as SA and ApoA-I likely underlies its sustained presence in plasma. Together, the docking data indicate that the superior absorption of the *Z*-isomers, particularly the 13*Z*-isomer, is driven by stronger interactions with uptake-related receptors (SR-BI and CD36), whereas their enhanced systemic stability can be attributed to efficient binding to plasma transport proteins (SA and ApoA-I) and reduced susceptibility to enzymatic metabolism *via* BCO2. Nevertheless, since several of the docking score differences among isomers were relatively small, for instance, between the 9*Z*- (−9.15 kcal mol^−1^) and 13*Z*-isomers (−9.07 kcal mol^−1^) in SR-BI, these trends should be interpreted with caution. Overall, these findings provide molecular-level insights into the distinct biological behavior of astaxanthin geometric isomers and underscore the functional significance of isomerization in modulating carotenoid bioavailability. It should be noted, however, that the docking results provide only supportive *in silico* evidence. Direct experimental validation, such as protein-binding assays or lipoprotein fractionation studies, will be required to confirm these computational predictions. In addition, this study was conducted using only male ICR mice; therefore, potential sex-dependent differences were not addressed. Future studies including female mice will be necessary to clarify whether the present findings apply across sexes.

## 4. Conclusions

This study provides the first comprehensive pharmacokinetic comparison of individually purified astaxanthin geometric isomers (*i.e.*, the all-*E*-, 9*Z*-, and 13*Z*-isomers) *via* both oral and intravenous administration in mice. The findings demonstrate that the 13*Z*-isomer possesses markedly superior bioavailability, systemic retention, and hepatic accumulation compared with the 9*Z*- and all-*E*-isomers. Non-compartmental and population pharmacokinetic analyses consistently revealed that the 13*Z*-isomer is characterized by low clearance, moderate distribution volume, and early absorption onset, whereas the 9*Z*-isomers exhibited rapid elimination, which is likely attributable to enzymatic degradation and *in vivo* isomerization. The isomer-specific pharmacokinetic behavior appears to result from multiple factors, including differences in solubility, lipoprotein-binding affinity, and enzymatic susceptibility. In particular, the strong affinity of the 13*Z*-isomer for plasma proteins (ApoA-I and SA) and transport receptors (CD36 and SR-BI), as indicated by molecular docking simulations, may underlie its favorable systemic profile. Conversely, the poor stability and metabolic vulnerability of the 9*Z*-isomer limit its bioavailability despite its rapid absorption. Molecular docking simulations further indicate that this vulnerability may be partly attributed to its higher affinity for BCO2, a carotenoid-cleaving enzyme involved in metabolism.

These results also help reconcile longstanding inconsistencies in the literature regarding the bioavailability of the *Z*-isomers of carotenoids such as β-carotene and lycopene, highlighting the need to consider individual isomer behavior rather than pooled *Z*-isomer fractions. From a translational perspective, the current findings indicate that targeting specific isomers, particularly (13*Z*)-astaxanthin, may enable the development of more effective dietary supplements and therapeutic formulations.

Overall, this work underscores the critical importance of isomer-specific pharmacokinetic profiling in carotenoid research and provides a foundation for future studies aiming to optimize the bioavailability and functional efficacy of geometric isomers in food and pharmaceutical applications.

## Author contributions

Antara Ghosh: data curation, investigation, validation, writing – review and editing; Yoshiharu Sawada: investigation, methodology, writing – review and editing; Kentaro Takahama: supervision, writing – review and editing; Yasuhiro Nishida: conceptualization, supervision, formal analysis, methodology, writing – review and editing; Masaki Honda: conceptualization, data curation, investigation, methodology, validation, funding acquisition, writing – original draft.

## Conflicts of interest

The authors declare that there are no conflicts of interest.

## Supplementary Material

RA-015-D5RA06060E-s001

## Data Availability

Data that supports the findings of this work is available from the corresponding author upon reasonable request. Supplementary information is available. See DOI: https://doi.org/10.1039/d5ra06060e.
