# Supplementary material for: Absorption characteristics and in vivo behavior of astaxanthin isomers: insights from the administration of highly purified (all-E)-, (9Z)-, and (13Z)-astaxanthin in male mice
Source: RSC Adv. 2025 Nov 10;15(51):43605–19. doi: 10.1039/d5ra06060e (PMC12598559; doi:10.1039/d5ra06060e)
Supplement: RA-015-D5RA06060E-s001 [file RA-015-D5RA06060E-s001.pdf]

Supplementary Information (SI) for RSC Advances.
This journal is © The Royal Society of Chemistry 2025

Supplementary Information

Absorption characteristics and *in vivo* behavior of astaxanthin isomers: Insights from the administration of highly purified (all-*E*)-, (9*Z*)-, and (13*Z*)-astaxanthin in male mice

Antara Ghosh [a], Yoshiharu Sawada [b], Kentaro Takahama [b], Yasuhiro Nishida [c,*], and Masaki Honda [a,d,*]

[a] *Department of Chemistry, Faculty of Science & Technology, Meijo University, 1-501 Shiogamaguchi, Tempaku-ku, Nagoya, Aichi 468-8502, Japan*

[b] *Technical Center, Nagoya University, Furo-cho, Nagoya, Aichi 464-8601, Japan*

[c] *Fuji Chemical Industries, Co., Ltd., 55 Yokohoonji, Kamiich-machi, Nakaniikawa-gun, Toyama 930-0405, Japan*

[d] *Graduate School of Environmental and Human Sciences, Meijo University, 1-501 Shiogamaguchi, Tempaku-ku, Nagoya, Aichi 468-8502, Japan*

***Corresponding authors**:

octopacy1978@gmail.com (Y. Nishida)

honda@meijo-u.ac.jp (M. Honda)

Table S1. Akaike's information criterion (AIC) and relative ΔAIC values for structural pharmacokinetic models tested for each astaxanthin isomer.

Isomer	Model	AIC	ΔAIC	CL (RSE%)	V (RSE%)	k_a (RSE%)	T_{lag} (RSE%)	Remarks
**all-*E***	1C	137.2	7.3	45 (67%)	102 (83%)	–	–	Poor fit, RSE > 50%
	**1C + T_{lag}**	**132.1**	**2.2**	**35.7 (48%)**	**75 (52%)**	**–**	**0.66 (38%)**	**Acceptable fit**
	1C + T_{lag} + EHC	131.4	1.5	34.5 (45%)	74 (49%)	–	0.63 (36%)	Slight improvement
	2C + T_{lag} + EHC	129.9	0	33.2 (42%)	69 (46%)	12.1 (65%)	0.58 (35%)	Best AIC, but poor k_a estimate
**9*Z***	1C	156.5	5.9	0.92 (75%)	3.2 (68%)	–	–	Unstable parameter estimates
	**1C + T_{lag}**	**150.6**	**0**	**0.74 (15%)**	**4.6 (12%)**	**0.57 (18%)**	**2.4 (22%)**	**Selected model**
	2C + T_{lag}	153.9	3.3	0.65 (35%)	3.5 (32%)	0.49 (33%)	2.3 (29%)	Overfitting trend
**13*Z***	1C	188.7	7.5	0.065 (61%)	2.6 (60%)	–	–	Poor precision
	**1C + T_{lag}**	**181.2**	**0**	**0.033 (22%)**	**1.9 (21%)**	**0.17 (25%)**	**0.12 (19%)**	**Selected model**
	2C + T_{lag}	183.7	2.5	0.031 (35%)	2.2 (34%)	0.19 (38%)	0.11 (29%)	No clear advantage
	1C + T_{lag} + lipid pool	182.4	1.2	0.030 (34%)	2.1 (33%)	0.15 (36%)	0.13 (28%)	Slightly better but unstable

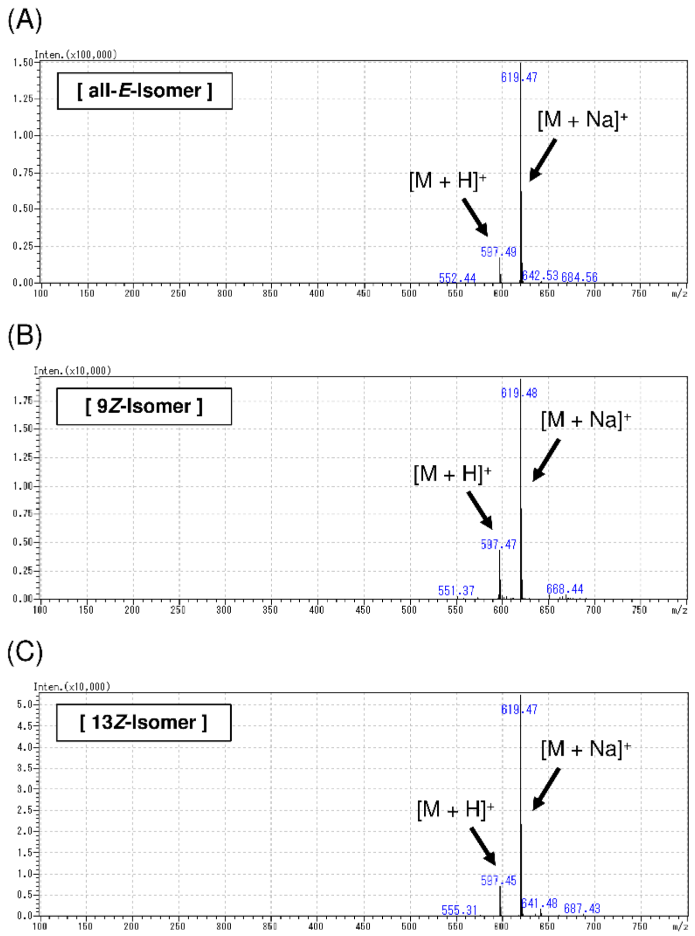

Fig. S1. Positive ion mode mass spectra of purified (A) (all-*E*)-, (B) (9*Z*)-, and (C) (13*Z*)-astaxanthin.

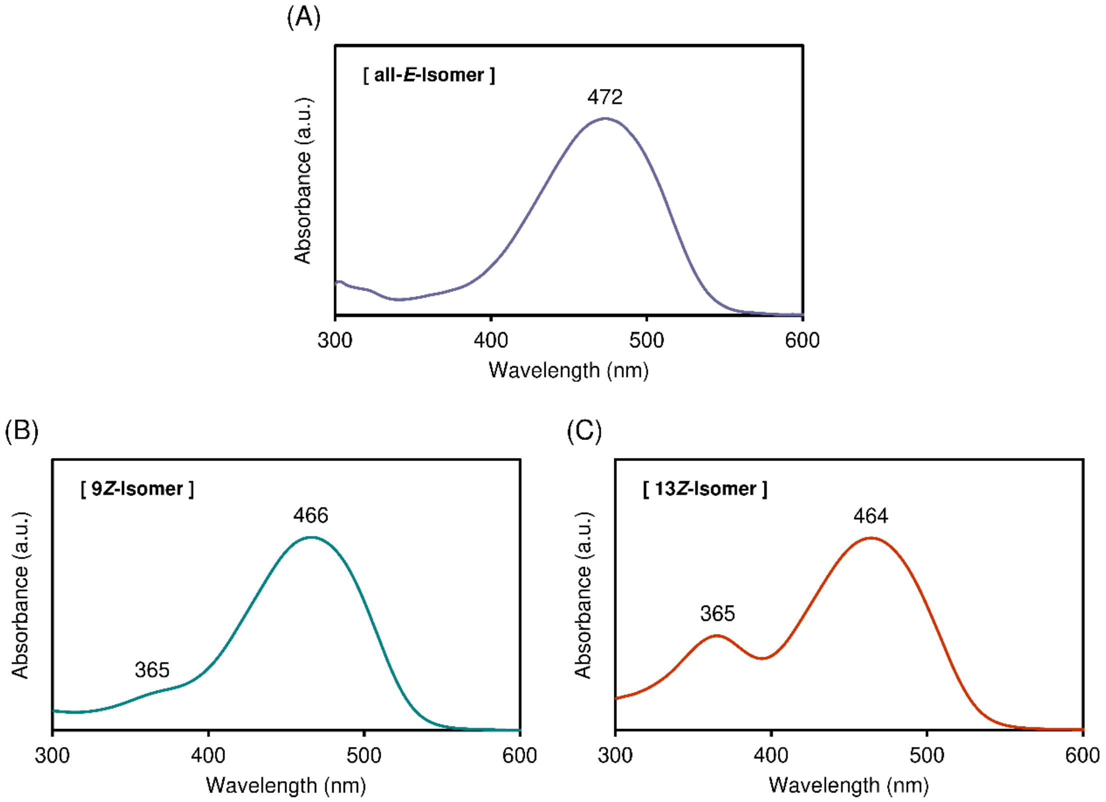

Fig. S2. Absorption spectra of purified (A) (all-*E*)-, (B) (9*Z*)-, and (C) (13*Z*)-astaxanthin.

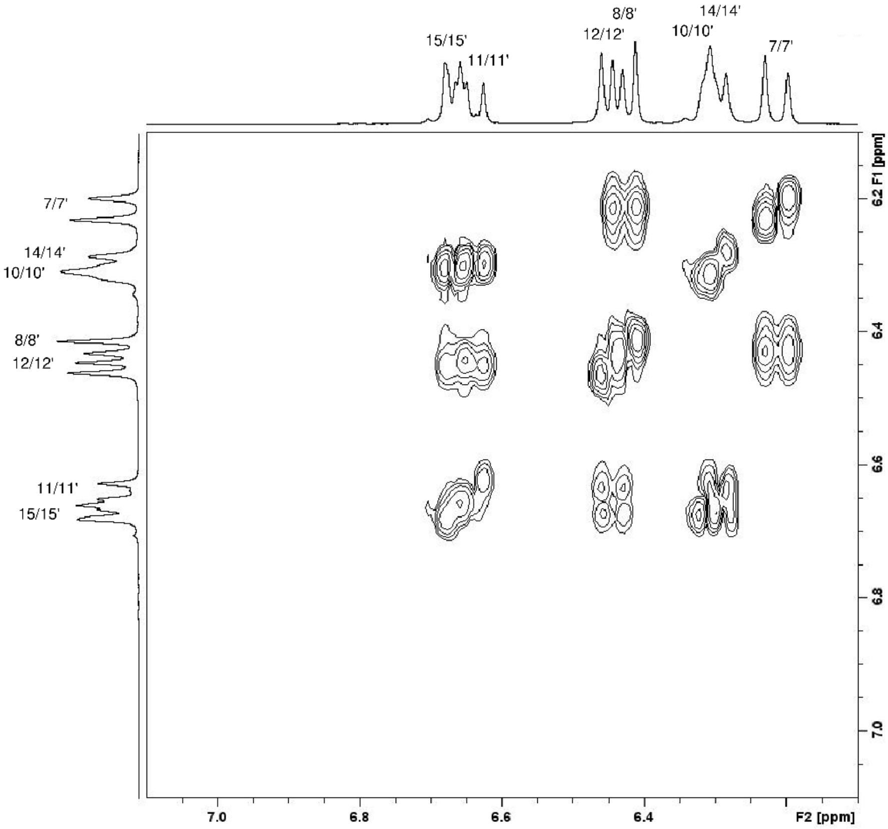

Fig. S3. ^1H–^1H COSY spectrum of (all-*E*)-astaxanthin in $CDCl_3$ in the range of 7.1–6.1 ppm, with peak assignments indicated.

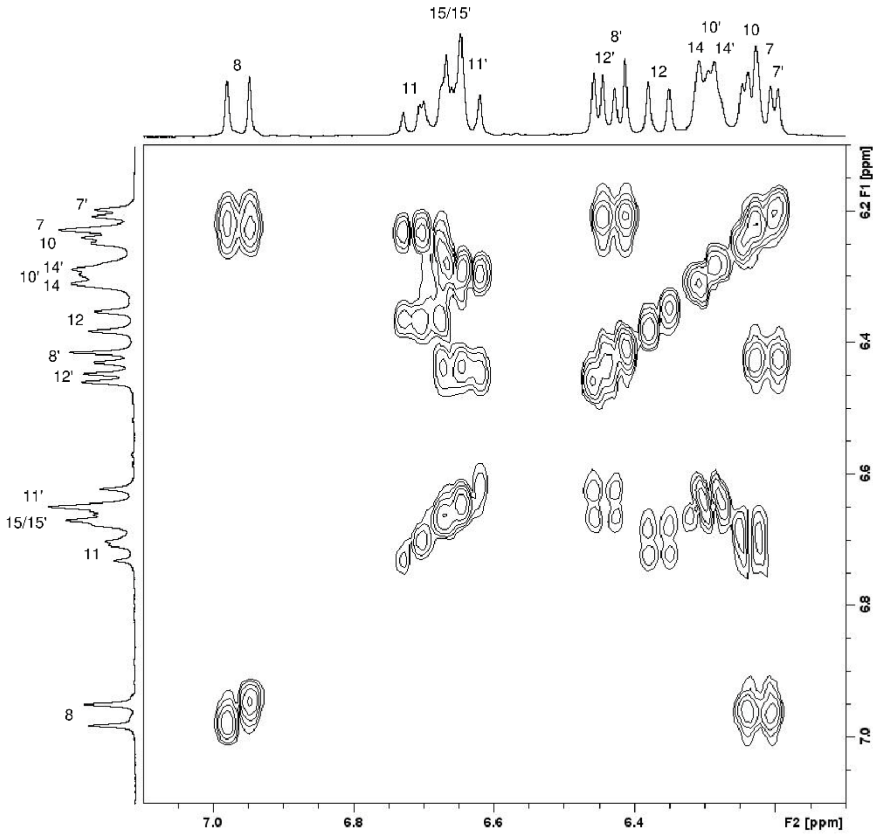

Fig. S4. ${}^{1}H$–${}^{1}H$ COSY spectrum of purified (9*Z*)-astaxanthin in $CDCl_3$ in the range of 7.1–6.1 ppm, with peak assignments indicated.

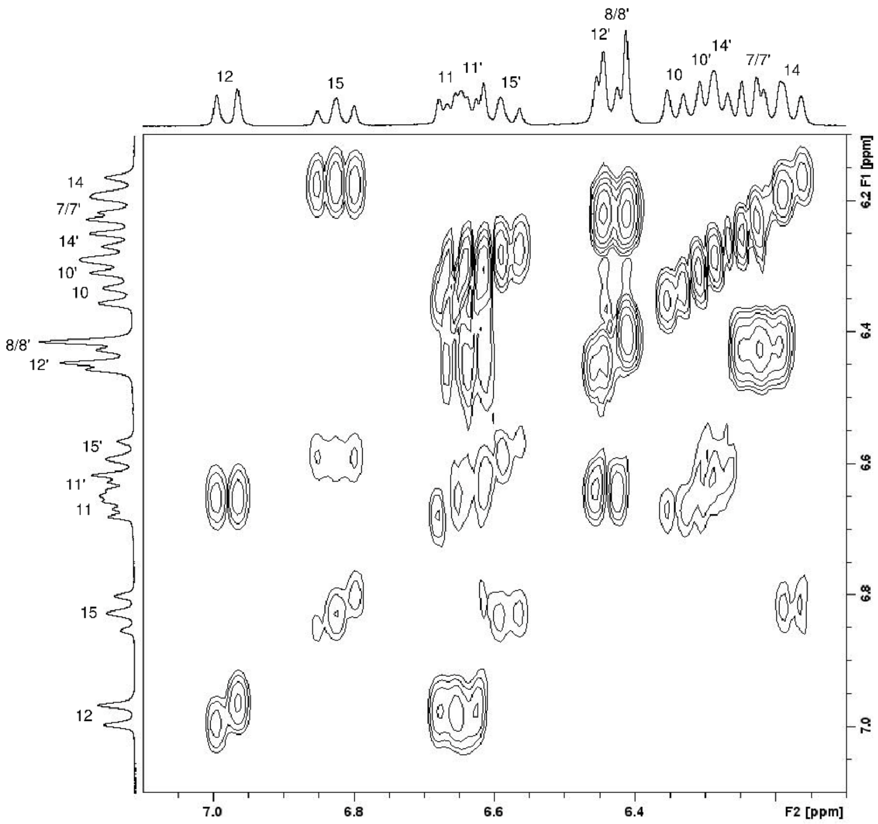

Fig. S5. ^{1}H-^{1}H COSY spectrum of purified (13*Z*)-astaxanthin in $CDCl_3$ in the range of 7.1–6.1 ppm, with peak assignments indicated.

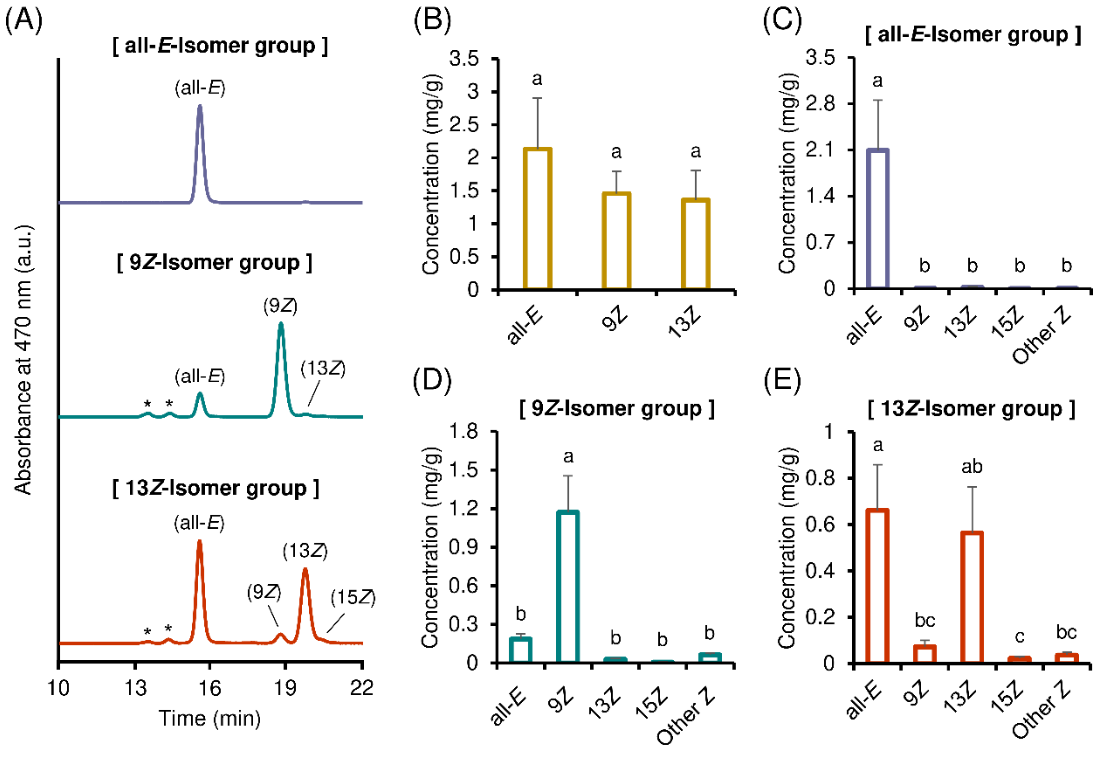

Fig. S6. (A) Normal-phase HPLC chromatograms of fecal samples after oral administration of astaxanthin isomers and (B) total and (C, D, E) each astaxanthin isomer concentration (mg/g) in fecal samples after oral administration of (C) (all-*E*)-, (D) (9*Z*)-, and (E) (13*Z*)-astaxanthin. Labels of (all-*E*), (9*Z*), (13*Z*), and (15*Z*) in (A) denote (all-*E*)-, (9*Z*)-, (13*Z*)-, and (15*Z*)-astaxanthin, respectively. The peaks marked with an asterisk (*) in (A) aare tentatively identified as astaxanthin *Z*-isomers. The means with different letters for each isomer in (B, C, D, E) are significantly different ($p < 0.05$). Error bars indicate the standard error.

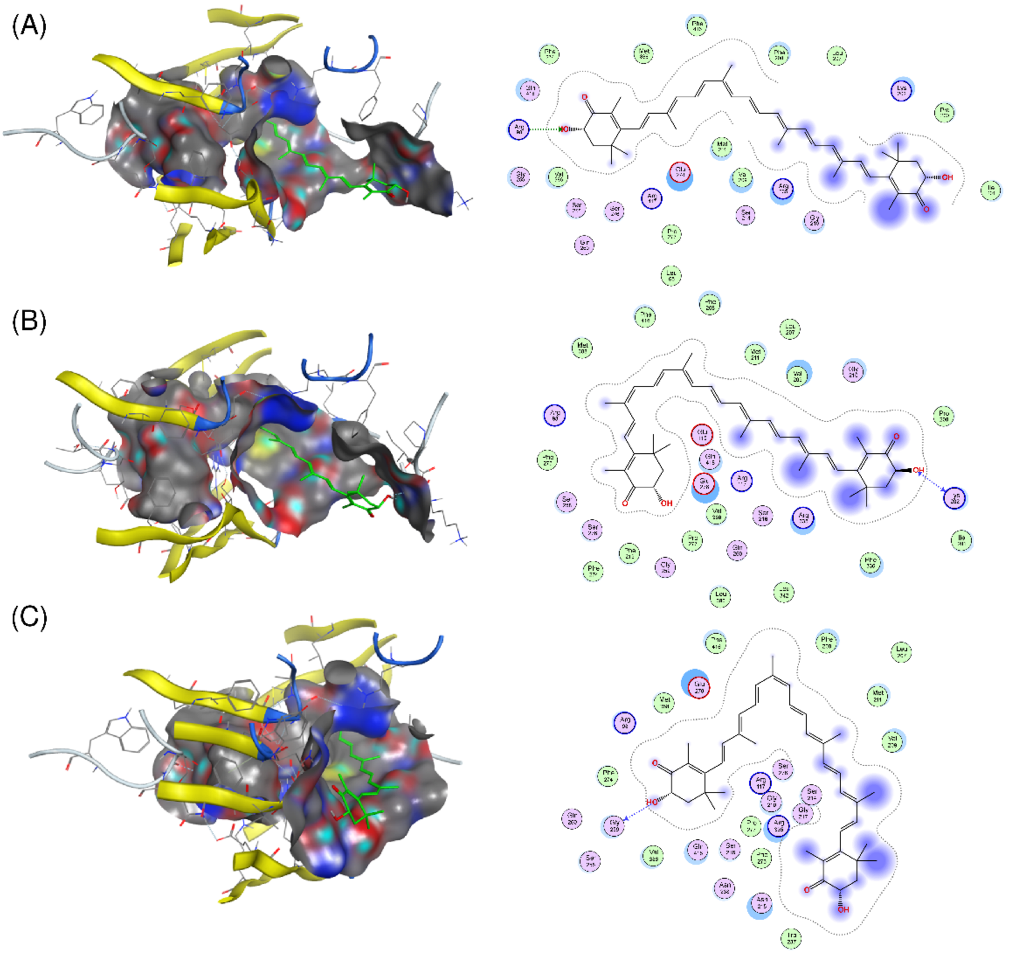

Fig. S7. The 3D (binding pocket surface representation binding pocket surface representation) and 2D interaction diagrams of scavenger receptor class B1 (SR-B1) with (A) (all-*E*)-, (B) (9*Z*)-, and (C) (13*Z*)-astaxanthin generated using MOE software. The 3D structures illustrate the ligand binding poses within the surface contour of the predicted binding pocket of SR-B1, while the 2D diagrams show the detailed molecular interactions with surrounding amino acid residues.

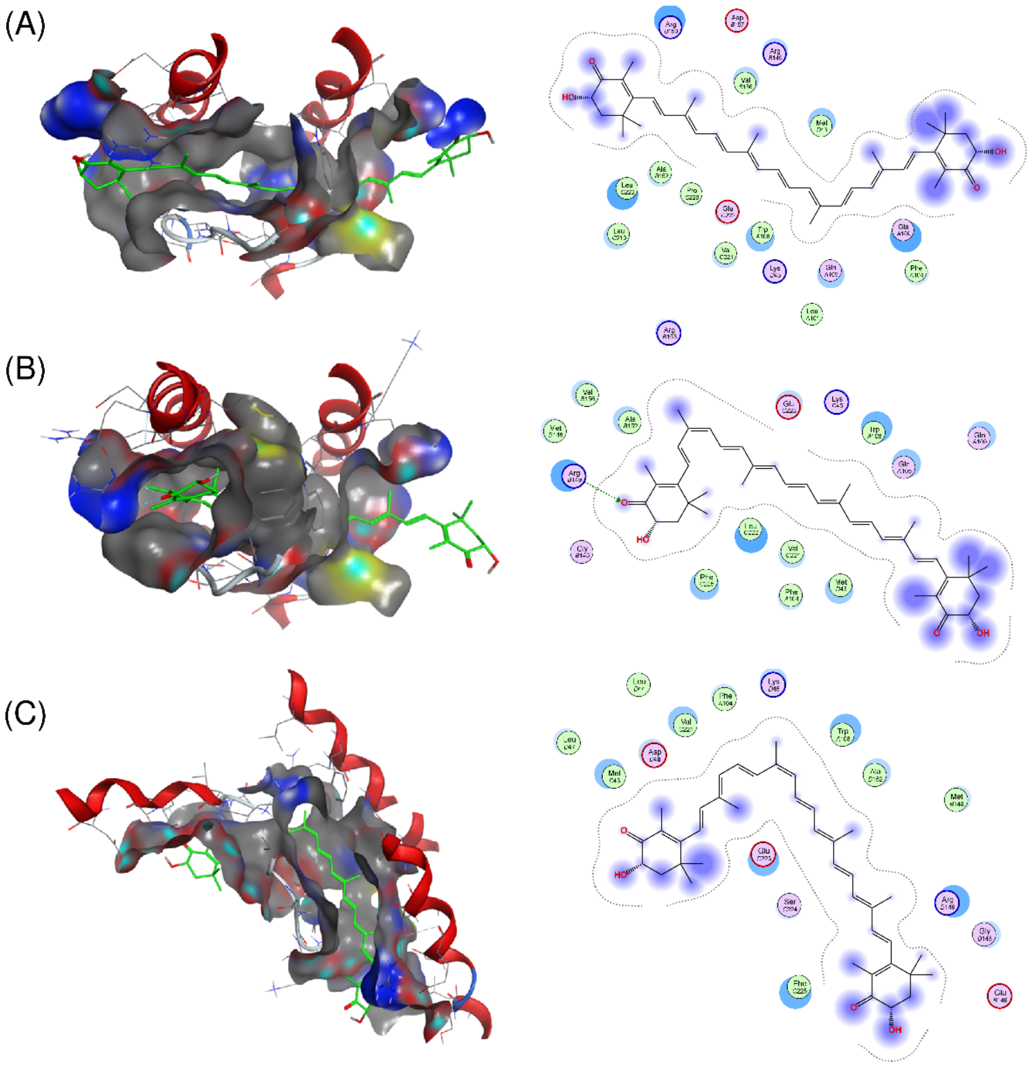

Fig. S8. The 3D (binding pocket surface representation) and 2D interaction diagrams of apolipoprotein AI (ApoA-I) with (A) (all-*E*)-, (B) (9*Z*)-, and (C) (13*Z*)-astaxanthin generated using MOE software. The 3D structures illustrate the ligand binding poses within the surface contour of the predicted binding pocket of ApoA-I, while the 2D diagrams show the detailed molecular interactions with surrounding amino acid residues.

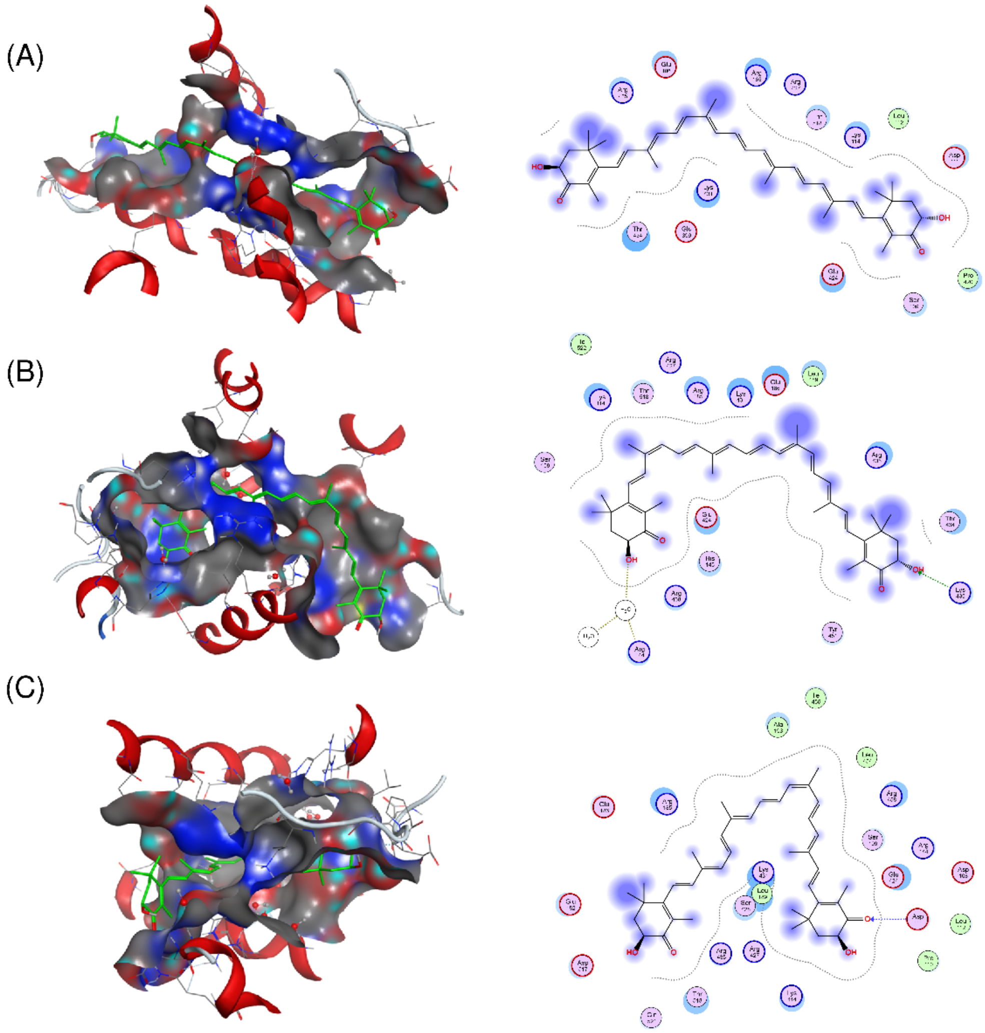

Fig. S9. The 3D (binding pocket surface representation) and 2D interaction diagrams of serum albumin (SA) with (A) (all-*E*)-, (B) (9*Z*)-, and (C) (13*Z*)-astaxanthin generated using MOE software. The 3D structures illustrate the ligand binding poses within the surface contour of the predicted binding pocket of SA, while the 2D diagrams show the detailed molecular interactions with surrounding amino acid residues.

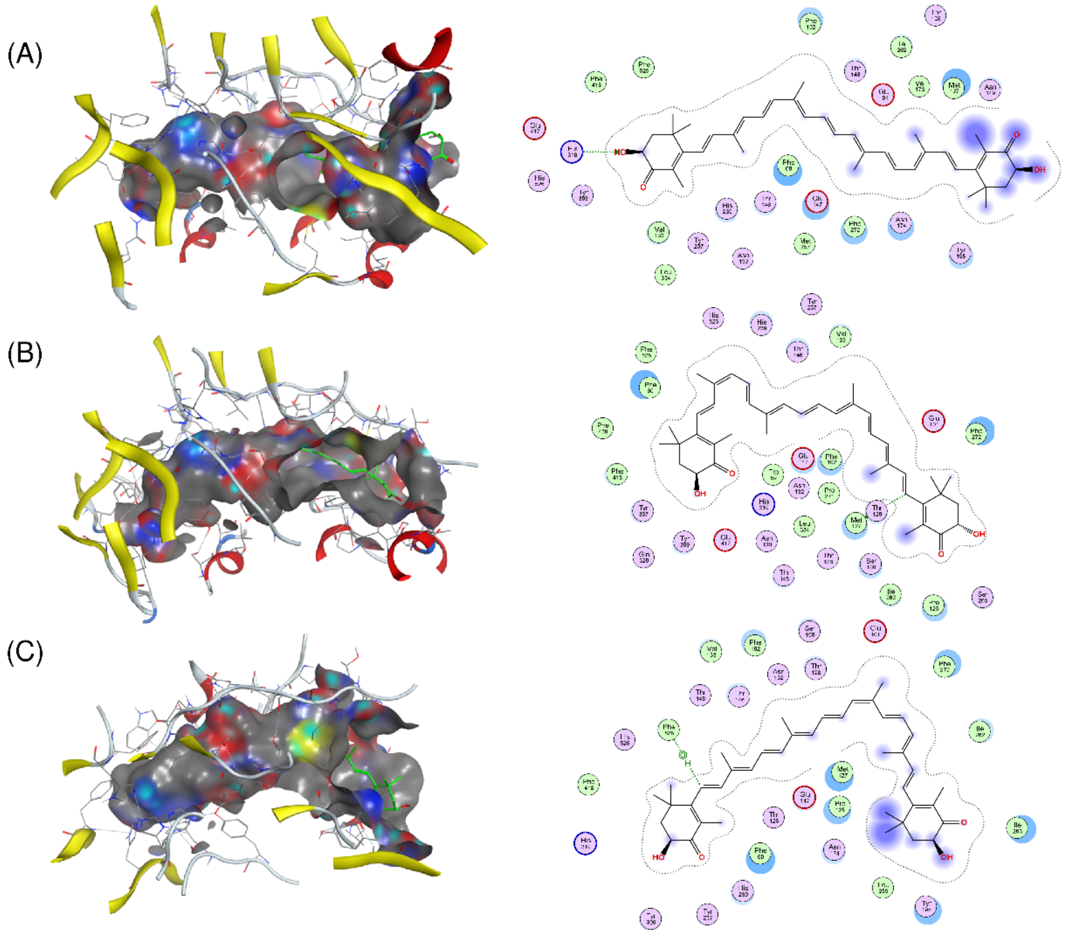

Fig. S10. The 3D (binding pocket surface representation binding pocket surface representation) and 2D interaction diagrams of β-carotene oxygenase-2 (BCO2) with (A) (all-*E*)-, (B) (9*Z*)-, and (C) (13*Z*)-astaxanthin generated using MOE software. The 3D structures illustrate the ligand binding poses within the surface contour of the predicted binding pocket of BCO2, while the 2D diagrams show the detailed molecular interactions with surrounding amino acid residues.
